# The role of mucosal immune dysregulation in the pathogenesis of immunoglobulin A nephropathy

**DOI:** 10.3389/fimmu.2026.1824906

**Published:** 2026-05-08

**Authors:** Yukako Ohyama, Yudai Tsuji, Hiroyuki Tezuka, Jan Novak, Kazuo Takahashi

**Affiliations:** 1 Department of Biomedical Molecular Sciences, Fujita Health University School of Medicine, Toyoake, Japan; 2 Department of Nephrology, Fujita Health University School of Medicine, Toyoake, Japan; 3 Department of Cellular Function Analysis, Research Promotion Headquarters, Fujita Health University School of Medicine, Toyoake, Japan; 4 Department of Microbiology, University of Alabama at Birmingham, Birmingham, AL, United States

**Keywords:** chemokine, cytokine, galactose-deficient IgA1, IgA nephropathy, mucosa-associated lymphoid tissue

## Abstract

Immunoglobulin A nephropathy (IgAN) is the most common form of primary glomerulonephritis worldwide with most patients progressing to kidney failure. Although its pathophysiology remains incompletely understood, deposition of IgA-containing immune complexes in the glomerular mesangium induces mesangial cell proliferation and overproduction of extracellular matrix components and cytokines and chemokines, that lead to glomerular injury. The properties of nephritogenic IgA1 include abnormal glycosylation of its polymeric forms and its capacity to bind IgG autoantibodies to form immune complexes. Nephritogenic IgA1 is thought to be secreted by B cells originating from or residing in mucosa-associated lymphoid tissues (MALT), such as gut-associated lymphoid tissues (GALT) and nasopharynx-associated lymphoid tissues (NALT). However, little is known how the immune abnormalities in MALT elevate the circulatory levels of nephritogenic IgA. This review summarizes fundamental insights into IgA production and its regulation in MALT in general, provides an overview of the immune abnormalities in the MALT of patients with IgAN relevant to the production of abnormally glycosylated IgA, and summarizes relevant emerging treatments tested in clinical trials.

## 1 Introduction

Immunoglobulin A nephropathy (IgAN), the most common form of primary glomerulonephritis, was first described by Berger and Hinglais in 1968 as a nephropathy characterized by mesangial IgA–IgG deposits (1, 2). The studied group of patients with recurrent hematuria exhibited mesangial hypercellularity and electron-dense deposits corresponding to mesangial IgA deposition in kidney biopsy specimens. These patients were typically young adults presenting with low-grade proteinuria and microscopic hematuria, which often worsened after upper-respiratory tract infections (1). This group of patients was later defined as “IgAN,” a chronic kidney disease that is now recognized as carrying a lifelong risk of progression to kidney failure (3–5).

From its earliest descriptions, IgAN has been considered a disorder in which abnormalities of mucosa-associated lymphoid tissues (MALT) contribute to disease pathogenesis. MALT is the principal inductive sites for mucosal immune responses and is widely distributed throughout the digestive, respiratory, and urogenital tracts, playing a crucial role as an expansive frontline network that strategically balances the elimination of diverse pathogens and maintains immune tolerance toward dietary antigens and the vast commensal microbiota (6–13). In this system, IgA molecules that are secreted into the lumen of these tracts, called secretory IgA, play an important role in immune exclusion and inclusion and maintain the homeostasis of microbiota and lymphoid tissue (14).

The “mucosa–kidney axis” hypothesis proposes that nephritogenic IgA molecules originate from mucosal immune sites (15). This concept is supported by several observations: disease flares frequently follow upper-respiratory tract infections or enteritis (16); patients with inflammatory bowel disease (IBD), including Crohn’s disease and ulcerative colitis (UC), exhibit an increased risk of developing IgAN (17–19), and secondary IgAN can also occur in individuals with celiac sprue (20); therapies targeting MALT, such as tonsillectomy and targeted-release budesonide (Nefecon), have demonstrated efficacy in patients with IgAN (21, 22); and polymeric and aberrantly glycosylated IgA species implicated in IgAN resemble IgA produced at mucosal surfaces (23). Furthermore, genome-wide association studies (GWAS) identified multiple susceptibility loci related to intestinal mucosal integrity and immune network regulation, providing additional support for this hypothesis (24).

Although the “mucosa–kidney axis” has gained significant attention, the precise cellular and molecular triggers within the gut-associated lymphoid tissues (GALT) and nasopharynx-associated lymphoid tissues (NALT) that shift IgA production toward a pathogenic profile are still being elucidated. This review aims to bridge the gap between basic mucosal immunology and the clinical manifestations of IgAN. We summarize the fundamental mechanisms of IgA synthesis, discuss the structural abnormalities of IgA identified through decades of research, and highlight how environmental factors, such as dysbiosis and Toll-like receptor (TLR) signaling, influence MALT, ultimately providing a rationale for emerging MALT-targeted therapeutic interventions.

## 2 Mechanisms of IgA production and its biological roles

IgA is the most abundant Ig isotype in the human body. Humans produce daily 40–60 mg of IgA per kg of body weight (25, 26). A fraction of this IgA enters circulation, whereas most of the produced IgA is in the secretions of various mucosal tissues, including the intestinal, respiratory, biliary, and genital tracts (25–27). In fact, at least 80% IgA antibody-secreting cells (IgA-ASCs) reside in the gut lamina propria (LP) (26, 28). Unlike serum IgA, which is predominantly monomeric, mucosal IgA assembles into dimeric IgA via the joining (J) chain (29). This J chain-containing IgA binds to the polymeric immunoglobulin receptor (pIgR) expressed on the basolateral surface of mucosal epithelial cells (30, 31), leading to transcytosis toward the apical surface, where pIgR is proteolytically cleaved into the secretory component (SC), which remains connected to IgA that is released into the lumen as secretory IgA (SIgA).

SIgA plays an important role in immune exclusion and inclusion within the intestinal lumen (14). In immune exclusion, SIgA promotes the clearance of luminal antigens, including pathogenic microorganisms, by blocking their access to epithelial receptors, entrapping them in the mucus. By coating the commensal bacteria in the lumen, SIgA allows the gut to prevent pathogen invasion without triggering inflammatory immune responses to pathogen-associated molecular patterns (PAMPs), such as TLR ligands. Moreover, SIgA promotes gut commensal agglutination, which limits their motility (32). Furthermore, SIgA cross-links the post-division daughter cells of dividing bacteria and prevents their separation by “enchainment” (33). Immune inclusion indicates pro-microbial activity mediated by SIgA and mucus, which promotes the growth of beneficial bacteria while preventing potentially harmful microorganisms from colonizing the same mucosal niches (34). In this manner, SIgA and the microbiota within the lumen are thought to interact with each other and maintain homeostasis of the bacterial flora.

The high dependency of IgA responses on intestinal colonization by commensal microorganisms reflects the complex relationship between IgA and the intestinal microbiota. This was demonstrated by comparing germ-free mice with specific pathogen-free mice. The germ-free mice have hypoplastic Peyer’s patches containing few germinal centers (GCs), IgA^+^ plasmablasts, and LP CD4^+^T cells (35). The close relationship between IgA production and the presence of microbiota has also been suggested during neonatal intestinal immune system development. Endogenous IgA production in human neonates begins several months after birth, and in mice, IgA-secreting plasma cells in the intestinal mucosal LP become apparent at around 4 weeks of age after weaning (36). Early-life exposure to microbial antigens during the neonatal period has been shown to shape the B-1 cell clonal repertoire that reacts to bacterial cell wall polysaccharide antigens, as well as the subsequent antibody responses (37, 38). Interestingly, the dynamics of IgA induction in infants depend on maternal SIgA; IgA-ASCs were detected earlier in mice not exposed to maternal SIgA via breast milk than in those who were exposed (39). Furthermore, Peyer’s patches play a major role in generating plasma cells that secrete IgA into maternal milk, and specific gut microbes are essential for properly programming maternal IgA production prior to lactation (40). Together, these findings suggest that maternal SIgA and the maternal gut immune–microbial environment cooperatively shape the timing and quality of IgA induction in offspring by regulating IgA-ASC development and programming.

Thus, the microbiota residing within MALT is a principal driver of IgA induction, and the IgA generated through this process exerts complex regulatory effects on the composition and homeostatic maintenance of the microbial community. These reciprocal interactions might influence IgA induction and microbiota establishment across generations.

Based on these findings, IgA induction is closely linked to microbial colonization, GC formation within the MALT, and proper IgA-ASC development and programming. Understanding the normal integrated process for IgA induction within the MALT is essential for a deep understanding of the dysregulated mechanisms underlying mucosal immunity-related IgAN. In this section, we focus on the four major steps involved in B-cell differentiation into IgA-ASCs and the subsequent IgA secretion into the mucosal lumen: antigen sampling in the intestine, V(D)J recombination and somatic hypermutation (SHM), IgA class switch recombination (CSR), and IgA-producing cell homing.

### 2.1 Antigen sampling

**Figure 1:**
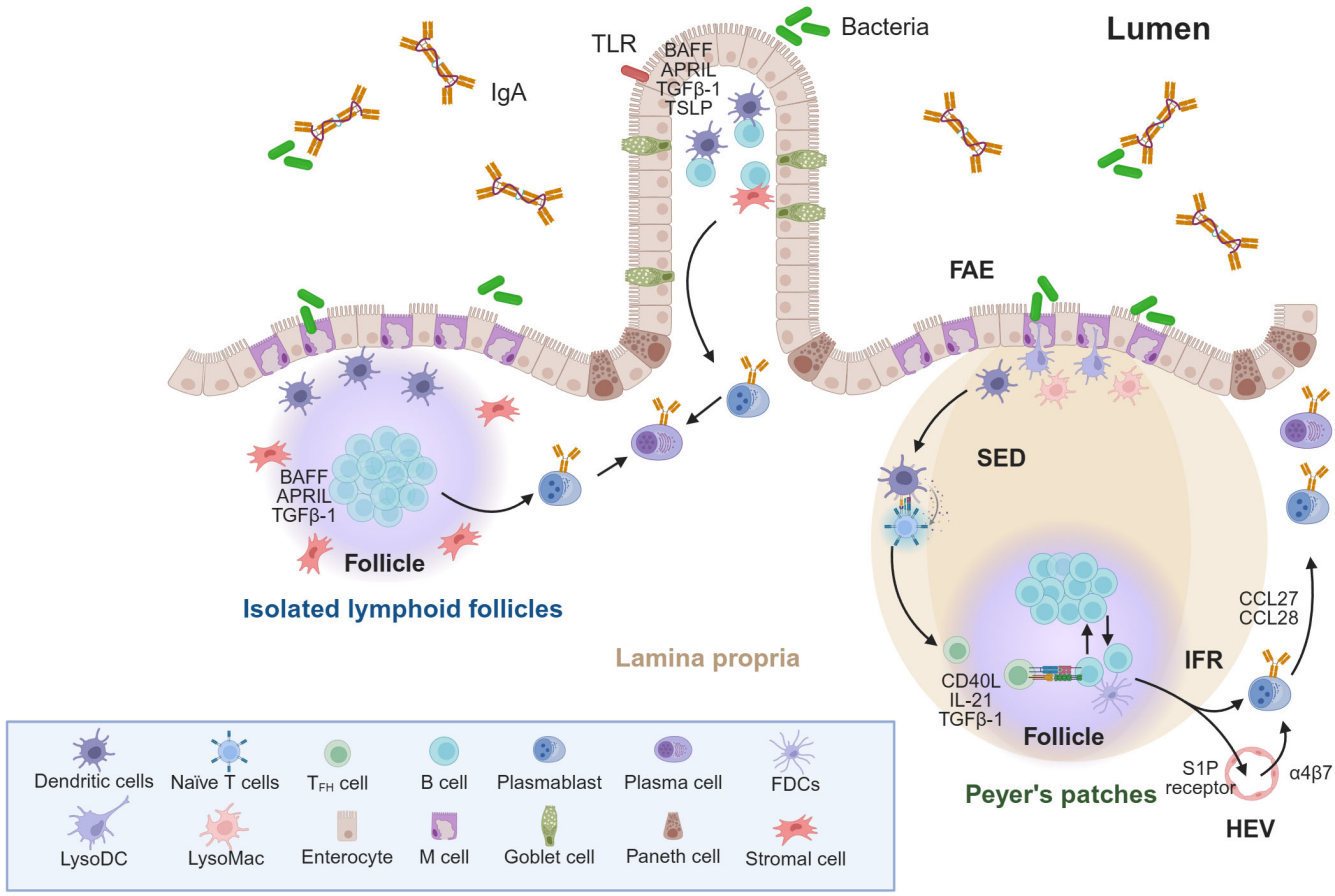
Schema of IgA induction systems in gut. This figure illustrates the orchestrated processes and the regulation of IgA^+^ cell dynamics within the intestinal immune system. Luminal antigens, such as bacteria and their components, are captured by M cells, and the captured antigens are presented by dendritic cells (DCs) to T and B cells within inductive sites, such as Peyer’s patches and isolated lymphoid follicles. LysoDC, LysoMac, and goblet cells have also been reported to capture luminal antigens. In the gut, B-cell activation and CSR to IgA are regulated by two distinct pathways: TD and TI. The TD pathway commonly occurs in the germinal centers of Peyer’s patches, whereas the TI pathway occurs primarily in the lamina propria and isolated lymphoid follicles. In the TD pathway, T_FH_ cells derived from dendritic cell-activated naïve T cells play an important role in the IgA CSR. CD40L–CD40 engagement under TGF-β (and IL-21 in humans) stimulation induce TD-IgA CSR on naïve B cells. Conversely, the TI-IgA CSR does not require T cells. TGF-β1 produced by antigen-captured DCs and a specialized stromal cell population and APRIL/BAFF produced by DCs, stromal cells, and epithelial cells induce TI-IgA CSR. TLR signaling activated by bacterial components also serves as a crucial trigger for BAFF/APRIL production, and TSLP secreted by epithelial cells boosts the production of DC-derived cytokines. Following IgA-ASC induction, plasmablasts emigrate from gut tissues via S1P gradients and undergo homing to the lamina propria, a process controlled by the expression of specific adhesion molecules and receptors such as CCR9/10, α4β7 integrin and S1P receptor. APRIL, A proliferation-inducing ligand; ASC, Antibody-secreting cell; BAFF, B-cell activating factor; CCL, C–C motif chemokine ligand; CD40L, CD40 ligand; CSR, Class switch recombination; FAE, follicle-associated epithelium; FDCs, follicular dendritic cells; HEV, high endothelial venule; IL-21, Interleukin-21; IFR, interfollicular region; LysoDC, lysozyme-expressing dendritic cell; LysoMac, lysozyme-expressing macrophage; M cells, microfold cells; SED, subepithelial dome; S1P, sphingosine-1-phosphate; TD, T-cell dependent; T_FH_, follicular helper T-cell; TGF-β1, transforming growth factor beta 1; TI, T cell-independent; TLR, toll-like receptor; TSLP, thymic stromal lymphopoietin.

Antigen sampling at the mucosal surface is a critical initiating step in mucosal immune responses, which are specialized to occur across the epithelial barrier. In GALT, this process is primarily mediated by microfold (M) cells localized within the follicle-associated epithelium (FAE) overlying Peyer’s patches in the small intestine and isolated lymphoid follicles (ILFs) embedded in the LP throughout the intestinal tract (**Figure 1**). Unlike classical enterocytes, M cells lack a thick glycocalyx and organized microvilli, allowing them to directly capture luminal antigens, including whole bacteria and viruses (41). Antigens captured by M cells are transported via transcytosis from the apical membrane to the basolateral pocket, where they are delivered to underlying dendritic cells (DCs) (41). Recent studies have reported that M cells can directly transfer antigens to B cells in the subepithelial dome (SED) of Peyer’s patches (42).

The upper-respiratory tract, including the palatine tonsils in humans, employs a distinct but functionally analogous strategy for antigen sampling (43, 44). The tonsillar surface is characterized by deep branched invaginations known as crypts, which significantly increase the surface area available for contact with inhaled or ingested antigens. The crypts also contain M-cell-like cells and DCs that actively transport antigens into the lymphoid follicles (44, 45).

In addition to these structures, lysozyme-expressing CX3CR1^+^ monocyte-derived phagocytes are mainly located in the SED and divided into at least two subsets: lysozyme-expressing DCs (LysoDCs) and lysozyme-expressing macrophages (LysoMacs). LysoDCs play an important role in sampling luminal particulate materials and intestinal microorganisms, including commensal bacteria and pathogenic bacteria, by extending the interepithelial projections through the FAE to the lumen (46–51). Because LysoDCs strongly express major histocompatibility complex class II (MHCII), they possess prominent antigen-presenting functions and prime naïve helper T cells to produce interferon (IFN) γ and IL-17 (52). Unlike LysoDCs, LysoMacs are CD4^+^ MHCII^lo^ cells with a slower renewal rate (49, 53). The antigen-sampling capacity of SED-resident LysoMacs are comparable to that of LysoDCs, whereas LysoDCs exhibit far stronger naïve Th cell-priming activity than SED-resident LysoMacs (48). Furthermore, small intestinal goblet cells deliver low-molecular-weight soluble antigens from the intestinal lumen to LP CD103^+^ DCs via goblet cell-associated antigen passage (54). These cellular and structural arrangements are particularly efficient at trapping particulate antigens and act as a robust first line of defense against mucosal pathogens.

### 2.2 B-cell differentiation to produce IgA

#### 2.2.1 V(D)J recombination and SHM

B cells diversify their antibody repertoire through three main genetic alterations that occur during two distinct phases of B-cell development: antigen-independent and antigen-dependent. In the antigen-independent phase, B-cell precursors in the bone marrow create many different antigen receptors by joining the variable (V), diversity (D), and joining (J) gene segments. This process, called V(D)J recombination, generates variable regions of heavy (H) and light (L) chains of immunoglobulins (55, 56). When the V_H_DJ_H_ and V_L_J_L_ exons are correctly assembled, the newly formed B cells expressing these chains as IgM and later as IgD migrate to secondary lymphoid organs, where they initiate antigen-dependent B-cell development (57). In the presence of antigens (in the context of antigen-presenting cells), mature B cells diversify their antibody repertoire through SHM and CSR. Both processes generally happen in the GC of secondary lymphoid organs and require activation-induced cytidine deaminase (AID), which converts cytosine to uracil in single-stranded DNA (58, 59).

SHM provide variants of B-cell receptors (BCRs) for the antigen-mediated selection of high-affinity antibody variants by introducing many point mutations, along with deletions or insertions, in the V_H_DJ_H_ and V_L_J_L_ exons (58–60). These mutations select B cells to produce antibodies with a high affinity for the antigen. SHM is markedly concentrated within the complementarity-determining regions (CDRs) of V-region exons (61). More antibody genes undergo extensive SHM in mucosal tissues than in peripheral blood or lymphoid tissues (62, 63). Highly mutated IgA genes develop through CD4^+^ T-cell- and GC-dependent processes and play a critical role in countering foreign mucosal pathogens and their toxins (64–66). In contrast, some low-affinity IgAs develop via T-cell- and GC-independent processes, possessing avidity properties that enable these antibodies to bind to commensal bacteria and regulate their composition (67, 68). When SHM occurs in low-affinity IgA, it is mostly random and may not increase affinity for commensal bacteria (69). CDR3 is generally considered the most critical region for antigen specificity, as it exhibits the highest degree of sequence diversity among the CDRs (70, 71). Several reports indicated that IgA1 in IgAN exhibits short CDR3 sequences, usually associated with low affinity, reduced diversity, and lack signs of affinity maturation (72–74). However, further studies are needed to confirm and extend these observations and their significance.

#### 2.2.2 IgA CSR

Mice and humans possess five Ig isotypes: IgM, IgD, IgG, IgA, and IgE (75), although they differ in subclasses of some Igs. Naïve B-cells display BCRs in both the IgM and IgD isotypes, that have identical antigen specificity within the same cell (76). This expression stems from the genomic organization in which the rearranged VDJ exon is followed by the heavy chain constant (C) regions *Cμ–Cδ* on the expressed *Igh* allele (76). After the *Cμ–Cδ* region, the *Igh* locus contains exons for secondary isotypes, as follows (75, 77):

**mouse: 5′-cµ–cδ–cγ3–cγ1-cγ2b–cγ2a–cϵ–cα-3′**

**human: 5′-cµ–cδ–cγ3–cγ1–cα1–cγ2–cγ4–cϵ–cα2-3′**

**Figure 2:**
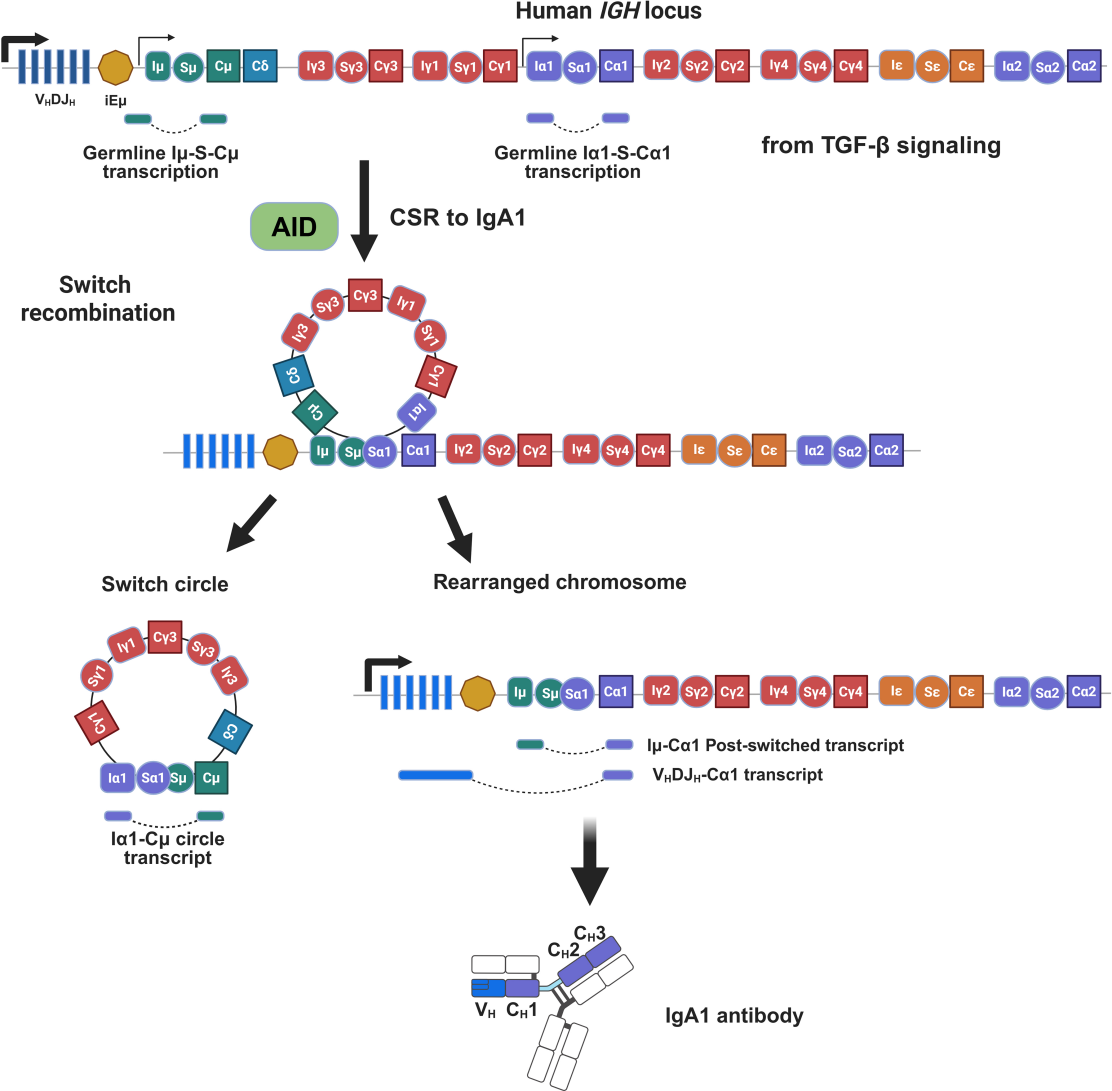
Class switch recombination of IgA1. This schematic illustrates the molecular mechanism of CSR to IgA1 within the human IGH locus. Following signaling that induces class switching to IgA, such as TGF-β, germline transcription is initiated, producing non-coding Iµ-S-Cµ and Iα1-S-Cα1 transcripts. This process facilitates switch recombination between the Sµ and Sα1 regions, leading to the excision of a DNA switch circle from this locus. The resulting chromosomal rearrangement brings the V_H_DJ_H_ unit into proximity with the Cα1 constant region gene. Ultimately, this genetic reorganization leads to synthesis of the IgA1 antibody. C, constant region; I, intervening region; S, switching region; AID, activation-induced cytidine deaminase; CSR, class switch recombination; C_H_, heavy chain constant region; V_H_, heavy chain variable region.

In CSR, the *Cμ–Cδ* regions are replaced by one of the downstream alternative isotypes (*Cγ*, *Cα*, or *Cϵ*). The secondary isotype induced by class switching is determined by the cytokine environment (56, 78, 79). For example, TGF-β predominantly leads to switching to IgA, at least *in vitro*, whereas IL-4 induces switching to both IgE and IgG1 (78). In response to cytokine stimulation, the corresponding germline transcripts (GLTs) are induced by unrearranged C-region genes. CSR is initiated when the transcription of these GLTs, a non-coding mRNA unit composed of an intervening (I)-region promoter, a switch (S)-region, and a downstream C-region gene, begins upstream of the intron promoter of the target S region (55, 60). This transcriptional activation renders the S-regions accessible, allowing AID to introduce DNA lesions. Subsequently, double-strand breaks are generated within the S-regions preceding each C-region gene (except *Cδ*), followed by their repair through non-homologous end-joining pathway (55, 60). During this process, the *IGH* locus recombines and the I-region DNA between the donor and acceptor S-regions is excised as a circular DNA by-product, completing the CSR reaction. The corresponding secondary isotype is expressed while maintaining antigen specificity (**Figure 2**). This allows antibodies to acquire distinct effector functions unique to each isotype. IgA CSR occurs through the highly complementary T-cell-dependent (TD) and -independent (TI) pathways.

#### 2.2.3 TD pathway

The TD pathway of IgA CSR commonly occurs within the GCs of Peyer’s patches (**Figure 1**). After sampling luminal antigens and microorganisms via FAE, TD-IgA synthesis is induced by antigen-bearing DC-stimulated CD4^+^ T cells, which subsequently differentiate into follicular helper T (T_FH_) cells expressing the transcription factor Bcl-6 and chemokine receptor C–X–C motif chemokine receptor 5 (CXCR5). CXCR5^+^ T_FH_ cells can migrate toward the follicle via C–X–C motif chemokine ligand 13 (CXCL13) produced by follicular dendritic cells (FDCs), where they differentiate into mature T_FH_ cells (80). The T_FH_ cells then interact with naïve B cells via CD40L–CD40 engagement, inducing TD-IgA CSR under TGF-β stimulation (81–84). TGF-β is constitutively produced by various cell types, including epithelial cells, DCs, and T cells. FDCs resident in Peyer’s patches produce large amounts of activated TGF-β1 upon TLR and retinoic-acid-receptor (RAR) stimulation (85). In addition, DCs can enhance TGF-β receptor expression on B cells by producing inducible nitric oxide (NO) synthase-derived NO (86). In humans, IL-21 cooperates with TGF-β to further promote IgA CSR and upregulate C–C motif chemokine receptor 10 (CCR10), while suppressing CXCR5, thereby driving IgA^+^ plasmablasts to exit the GC and migrate toward mucosal tissues that express the CCR10 ligands, such as C–C motif chemokine ligand 27 (CCL27) and CCL28 (87). In patients with tonsillitis, most IgA^+^ cells within the GCs express CCR10, and those located in the subepithelial region show higher CCR10 expression than their counterparts in the GCs, indicating that, after induction in the GCs, these IgA^+^CCR10^+^ cells could migrate out of the GCs to populate mucosal surfaces (87).

#### 2.2.4 TI pathway

The TI pathway of IgA production was identified based on the observation that CD40-deficient mice generate normal levels of IgA-ASCs in the intestinal LP despite their inability to form GCs (66). In these mice, the number of mutations in the IgV regions and SHM frequency are markedly reduced than those in wild-type mice, producing polyreactive IgA with low antigen affinity. These findings suggest that IgA induced through the TI pathway possesses lower antigen affinity than IgA generated through the TD pathway (88, 89). Under homoeostatic conditions, IgA is primarily produced by plasma cells localized in the small intestinal LP via the TI pathway triggered by most commensal bacteria and their products, such as TLR ligands, yielding SIgA with low affinity and broad reactivity with common microbial antigens (90). These antibodies have a limited capacity to mediate the attachment of various bacterial species to the host epithelium (90).

TI pathway induction relies on B-cell-activating factor (BAFF) and a proliferation-inducing ligand (APRIL), which are cytokines belonging to the tumor necrosis factor (TNF) superfamily of DCs, stromal cells, and epithelial cells (14, 79, 89) (**Figure 1**). Antigens that trigger the TI pathway differ functionally and structurally from those activating the TD pathway. The TD pathway is mainly activated by protein antigens, whereas the TI pathway is primarily activated by non-protein antigens, which are mainly classified as Type-1 TI (TI-1) and Type-2 TI (TI-2) antigens. TI-1 antigens may activate B cells through signaling via TLRs that recognize microbial products such as lipopeptides, lipopolysaccharide (LPS), microbial cytidine-phosphate guanosine (CpG) DNA, and viral RNA proteins (91, 92). TI-2 antigens typically exhibit repetitive structures, such as bacterial capsular polysaccharides, that can crosslink BCRs and provide persistent signals to B cells through the Bruton’s tyrosine kinase (BTK), a non-receptor tyrosine kinase that plays a pivotal role in B-cell development, activation, and proliferation (92–94).

The TI pathway primarily occurs in the intestinal LP or ILFs (79). Within these tissues, B-cell activation relies heavily on DC- and TLR-mediated signaling (89). In ILFs, luminal antigens are taken up via M cell- or DC-dependent pathways. A specialized stromal cell population and antigen-captured DCs produce TGF-β, which is essential for IgA CSR (79). In addition, lymphoid tissue inducer (LTi) cells help organize the follicle structure and supply cytokines that further support IgA induction (89). In the LP, both classical/conventional DCs and plasmacytoid DCs (pDCs) release BAFF and APRIL. TLR ligand-stimulated epithelial cells also produce both BAFF and APRIL, and secrete thymic stromal lymphopoietin (TSLP), which boosts DC-derived cytokine production (95, 96). A specialized DC subset further amplifies BAFF and APRIL production via NO production (86).

**Figure 3:**
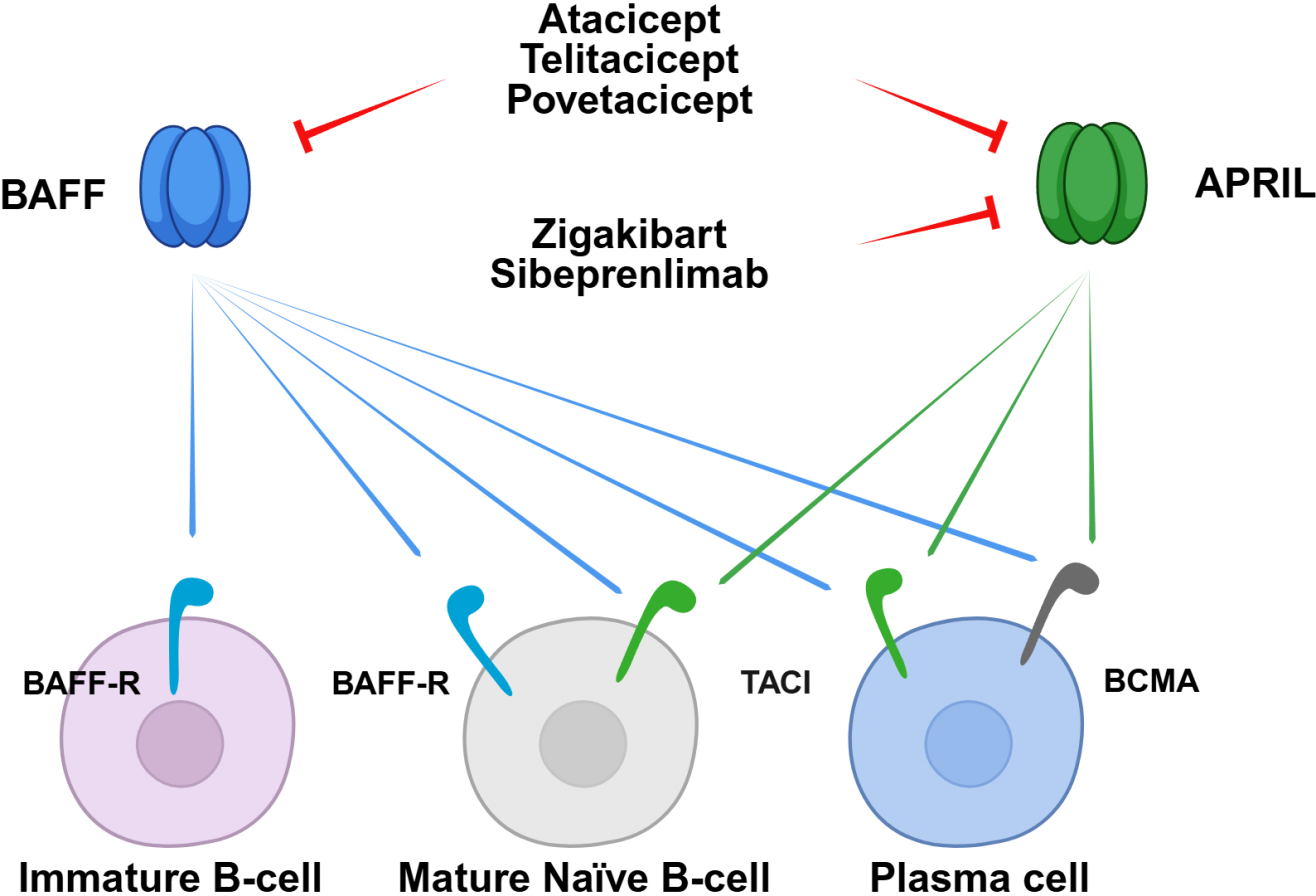
BAFF/APRIL system and therapeutic targets of novel BAFF/APRIL inhibitors. Schematic illustration of the therapeutic targeting of BAFF and APRIL signaling pathways across different stages of B-cell differentiation and maturation. The maturation process from immature B cells to mature naïve B cells, and eventually to plasma cells, is regulated by the key ligands BAFF and APRIL. These ligands interact with their respective receptors, including BAFF-R, TACI, and BCMA, to promote cell survival and differentiation. Various inhibitors are categorized based on their ligand specificity and binding mechanisms: Sibeprenlimab and zigakibart are monoclonal antibodies that specifically neutralize APRIL, while povetacicept, atacicept, and telitacicept function as standard TACI–Fc fusion proteins that dually inhibit both BAFF and APRIL. Povetacicept is an engineered TACI–Fc fusion protein with enhanced potency and inhibitory activity. Collectively, these therapeutic interventions aim to modulate aberrant B-cell responses and reduce pathogenic IgA1 production. APRIL, a proliferation-inducing ligand; BAFF, B-cell activating factor; BCMA, B-cell maturation antigen; BAFF-R, B-cell activating factor receptor; TACI, transmembrane activator and calcium modulator and cyclophilin ligand interactor.

BAFF and APRIL signaling is mediated by binding to the BAFF receptor (BAFF-R; also known as TNF receptor superfamily member 13C), B-cell maturation antigen (BCMA; also known as TNF receptor superfamily member 17), and transmembrane activator and cyclophilin ligand (TACI; also known as TNF receptor superfamily member 13 B) (**Figure 3**). APRIL binds to BCMA and TACI, whereas BAFF binds to BCMA, TACI, and BAFF-R (97). The expression levels of these receptors correlate with B-cell differentiation stage (98). BAFF-R is expressed during early B-cell development, including in immature B cells, whereas BCMA and TACI are predominantly expressed in the late stages of differentiation, such as in plasmablasts and plasma cells (98).

### 2.3 Migration and homing of IgA-producing cells

The immune system is a dynamic and highly organized network of various types of cells. Some of these cells can migrate to specific organs, depending on the physiological or pathophysiological situation. Successful recruitment of lymphoid cells to the target tissue requires chemokines, selectins, and integrins, and the relevant signaling pathways.

**Figure 4:**
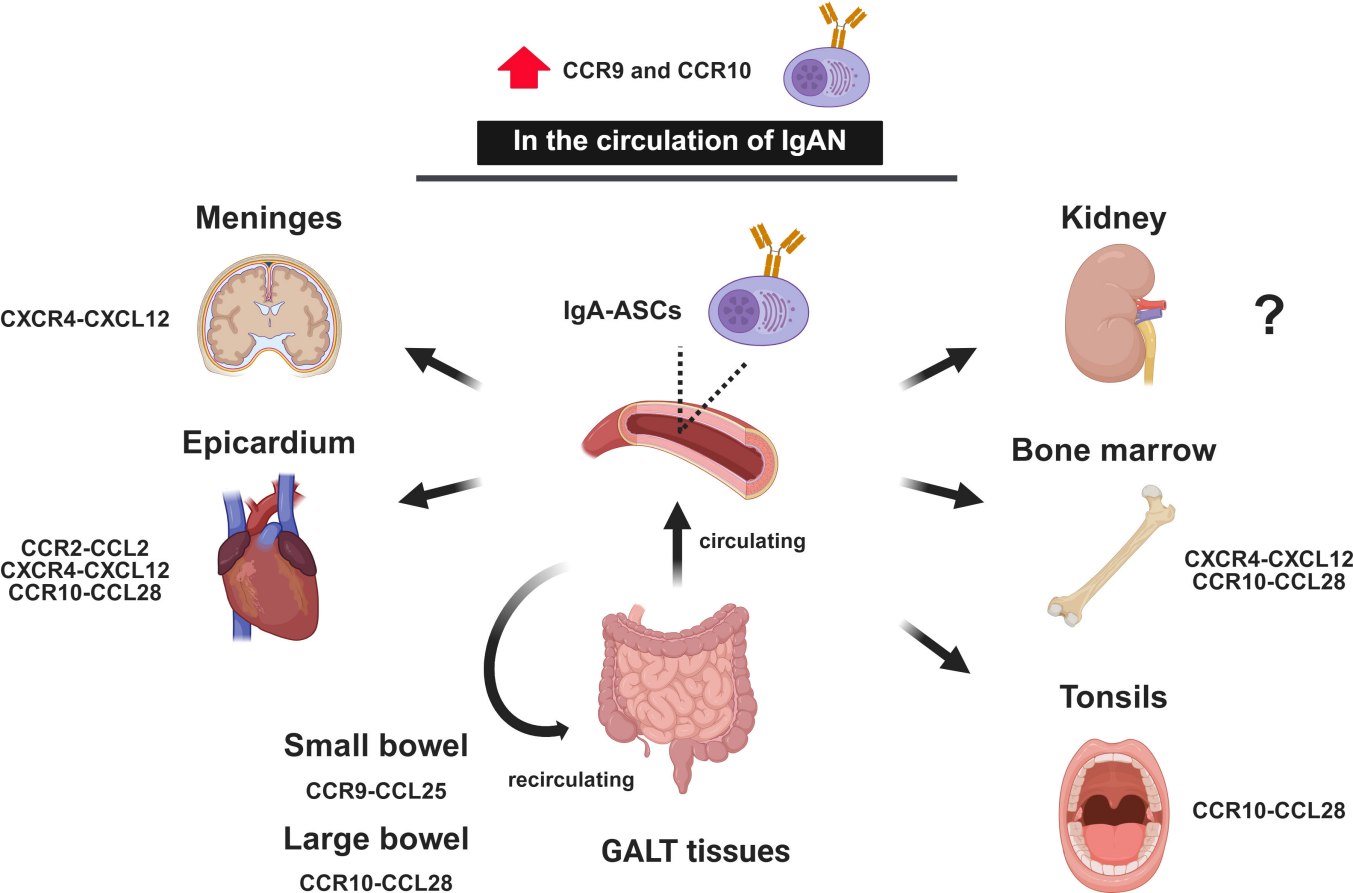
IgA-producing cell homing. Schematic illustration of the systemic migration and homing patterns of ASCs originating from GALT. Following activation in GALT, IgA-ASCs enter the circulation and are recruited to various mucosal and non-mucosal effector sites. Distinct combinations of chemokines and receptors orchestrate tissue-specific homing. For instance, migration to the small and large bowels/tonsils is primarily mediated by the CCR9–CCL25 and CCR10–CCL28 axes, respectively. Furthermore, the diagram depicts the recruitment of IgA-ASCs to extraintestinal organs, including the meninges, kidney, bone marrow, and epicardium, facilitated by signaling pathways such as CCR2–CCL2, CXCR4–CXCL12, and CCR10–CCL28. These homing pathways highlight the role of IgA-ASCs in providing broad protective immunity and maintaining homeostasis across multiple anatomical compartments through circulating and recirculating mechanisms. Notably, in IgAN, the frequency of circulating IgA-ASCs that express CCR9 and CCR10 increases. GALT, gut-associated lymphoid tissue; IgA-ASCs, IgA antibody-secreting cells; CCR, C–C motif chemokine receptor; CXCR, C–X–C motif chemokine receptor; CXCL, C–X–C motif chemokine ligand; CCL, C–C motif chemokine ligand; IgAN, IgA nephropathy.

IgA^+^ plasmablasts induced in GALT enter the systemic circulation and selectively return to the intestinal mucosa. This migration system is called “homing,” which is tightly regulated by homing receptors on plasmablasts and their ligands and/or counter-receptors on the intestinal tissues. IgA^+^ plasmablasts expressing sphingosine 1 phosphate (S1P) receptors sense S1P gradients to migrate from GALT into circulation (99). Circulating IgA^+^ plasmablasts then migrate into the intestinal LP through the interactions of CCR9–CCL25, CCR10–CCL28, and integrin α4β7–mucosal addressin cell–adhesion molecule 1 (MAdCAM-1). CCL25 and CCL28 are chemokines expressed by the intestinal epithelial and endothelial cells, whereas MAdCAM-1 is a key ligand for the integrin α4β7 and is expressed in the intestinal postcapillary venules (100–102). In humans, all circulating IgA-ASCs express CCR10 and 55% co-express β7 integrin, but not CCR9 at steady state (103). In human bone marrow, IgA-ASCs account for nearly 40% of plasma cells at steady state. Almost all bone-marrow plasma cells express CXCR4, which interacts with CXCL12 expressed by bone marrow stromal cells and gut epithelium. Approximately 40% and 30% of bone-marrow IgA-ASCs express CCR10 and integrin β7, respectively (103). These findings suggest that mucosal tissue-derived IgA-ASCs are predominant in the bloodstream and in a subset of bone-marrow niches, and further indicate that they retain the capacity to migrate back to mucosal sites (**Figure 4**).

Recent advancements in experimental technologies and bioinformatics have enabled rapid exploration of diverse biological processes at a single-cell resolution. Two studies showed that meninges and epicardium are significant destinations for gut-derived IgA^+^ cells. Mouse models of amyloid β-driven Alzheimer’s disease revealed that neuroinflammation reshapes the colonic immune landscape, triggering the recruitment of gut-derived IgA^+^ cells to the meninges (104). This specific migratory axis is primarily driven by the CXCR4–CXCL12 signaling pathway, which facilitates the relocation of intestinal effector cells to the cranial interface. Furthermore, spatially resolved multiomics of human cardiac niches indicated that the epicardium is a distinct destination for IgA-producing cells (105). The accumulation and maintenance of these cells within the epicardial niche is orchestrated by a coordinated set of chemokine interactions. Specifically, the CCL2–CCR2, CXCL12–CXCR4, and CCL28–CCR10 pairs of ligands-receptors have emerged as the key molecular candidates governing this process.

IgA-ASCs are present within the renal tubulointerstitial regions in both IgAN animal models and human patients (106). Notably, the number of CD138^+^ cells relative to glomeruli in biopsy samples positively correlate with serum creatinine levels and the degree of proteinuria in patients with IgAN (106). These findings suggest a potential link between local plasma cell infiltration and disease severity. Conversely, in mouse models of multiple sclerosis (MS), a neuroimmune disorder, IgA-ASCs infiltrate the central nervous system (CNS) and exert protective effects that is attributed to IL-10 production by these cells (107). This is further supported by the clinical observation that atacicept (TACI-Ig, described later) treatment in patients with relapsing-remitting MS dose-dependently increases disease activity and occurrence of serious adverse events, suggesting a beneficial role for CNS-infiltrating IgA-ASCs in MS (108). Consequently, investigating the homing mechanisms, functional roles, and molecular signatures of IgA-ASCs in the kidney during glomerulonephritis is indispensable for advancing our understanding of IgAN pathogenesis (**Figure 4**).

## 3 The characteristics of serum IgA in patients with IgAN—Key insights gathered over three decades

IgAN is characterized by the deposition of IgA-containing immune complexes in the glomerular mesangial areas. The deposits contain exclusively IgA1 subclass (109). Although serum IgA levels are elevated in approximately 50% patients with IgAN, mesangial IgA deposition does not occur at high frequency in other conditions associated with increased serum IgA, such as IgA myeloma, except in specific cases with abnormal glycosylation (110, 111). Furthermore, mesangial IgA deposition disappeared after kidney transplantation from a donor with subclinical IgAN to a non-IgAN patient (112). Conversely, IgAN histologically recurs in up to 60% patients after kidney transplantation (113). In addition, IgA deposits were not detected following bone marrow transplantation in patients with IgAN (114). These pieces of evidence support the theory that a fundamental abnormality in IgAN resides in the characteristics of IgA itself rather than in the intrinsic abnormalities of the kidneys. In this section, we review the characteristics of circulating IgA in patients with IgAN over the past 30 years, with reference to the basic human IgA structure (**Table 1**).

**Table 1:** Characteristics of IgA molecules in patients with IgA nephropathy.

Subjects	Source of IgA	Characteristics	Reference
Serum IgA concentration	Serum	Higher in most patients with IgAN compared to healthy controls.	Tomino et al., 2000 (J Clin Lab Anal) (121) Moldoveanu et al., 2007 (Kidney Int) (122) Harper, et al., 1994 (Am J Kidney Dis) (123)
IgA subclass	Serum	Serum IgA1 levels were higher in patients with IgAN compared to healthy controls. Ratio of IgA1 to IgA was higher in patients with IgAN compared to healthy controls.	van den Wall Bake et al., 1988 (Clin Exp Immunol) (124) Peterman et al., 1991 (Am J Kidney Dis) (125) Harper, et al., 1994 (Am J Kidney Dis) (123) Chui et al., 1991 (J Clin Immunol) (127)
		Serum IgA and IgA1 levels were higher in patients with IgAN, however, the ratio of serum IgA1 to total IgA was identical.	Chen et al., 1991 (Nephrol Dial Transplant) (126)
	Bone marrow	In bone-marrow cultures, both monomeric and polymeric IgA1 were elevated in patients with IgAN compared with healthy controls. The ratio of polymeric IgA1 to total IgA1 was comparable between the two groups.	van den Wall Bake et al., 1989 (Kidney Int) (128)
		In bone marrow, percentage of IgA plasma cells and IgA plasma cells bearing subclass IgA1 was greater in patients with IgAN compared to healthy controls.	Harper, et al., 1994 (Am J Kidney Dis) (123)
Predominance of λ light chain	Serum	Serum IgA1 κ/λ ratio was significantly lower in patients with IgAN compared to healthy controls.	Chen et al., 1991 (Nephrol Dial Transplant) (126) Chui et al., 1991 (J Clin Immunol) (127)
	Glomeruli	λ light chain deposition is dominant in glomeruli of patients with IgAN.	Lai et al, 1986 (Am J Clin Pathol) (129) Jennette et al., 1988 (Am J Kidney Dis) (130) Ravipati et al., 2022, (Kidney Int Rep) (132) Rizk et al., 2023 (J Clin Med) (131)
IgA molecular weight	Serum	pIgA is increased in serum of patients with IgAN compared to healthy controls.	Trascasa et al., 1980 (Clin Exp Immunol) (133) Valentijn et al., 1984 (Kidney Int) (134) Feehally et al., 1986 (Kidney Int) (135)
		pIgA is increased with a concomitant increase of mIgA of similar magnitude.	Newkirk et al., 1983 (J Immunol) (138)
		pIgA is not increased.	Woodroffe et al., 1980 (Kidney Int) (136) Lesavre et al., 1982 (Clin Exp Immunol) (137)
	Bone marrow	The ratio of pIgA1 to total IgA1 was not increased in bone-marrow culture.	van den Wall Bake et al., 1989 (Kidney Int) (128)
IgA glycosylation feature	Serum	Higher affinity for HAA.	Allen et al., 1995 (Clin Exp Eimmunol) (145) Moldoveanu et al., 2007 (Kidney Int) (122) Shimozato et al., 2008 (Nephrol Dial Transplant) (146)
		pIgA shows four-fold higher affinity with HAA than mIgA.	Oortwijn et al., 2006 (J Am Soc Nephrol) (139)
		Alterations in galactosylation, sialylation, bisection, biantennary, and fucosylation in *N*-glycans, and decreased sialylation of *O*-glycan was observed in IgA of patients with IgAN.	Dotz et al., 2021 (J Am Soc Nephrol) (150)
		pIgA in patients with IgAN showed decreased number of GalNAc residues in *O*-glycopeptide.	Yu et al., 2021 (Nephrol Dial Transplant) (151)
		Plasma or serum IgA showed decreased number of GalNAc residues in *O*-glycopeptide.	Nakazawa et al., 2019 (Biochem Biophys Res Commun) (152) Zhang et al., 2022 (Front Mol Biosci) (153) Ohyama et al., 2022 (iScience) (154)

IgAN, IgA nephropathy; pIgA, polymeric IgA; HAA, *Helix aspersa* agglutinin; mIgA, monomeric IgA; GalNAc, *N*-acetylgalactosamine.

### 3.1 Human IgA

**Figure 5:**
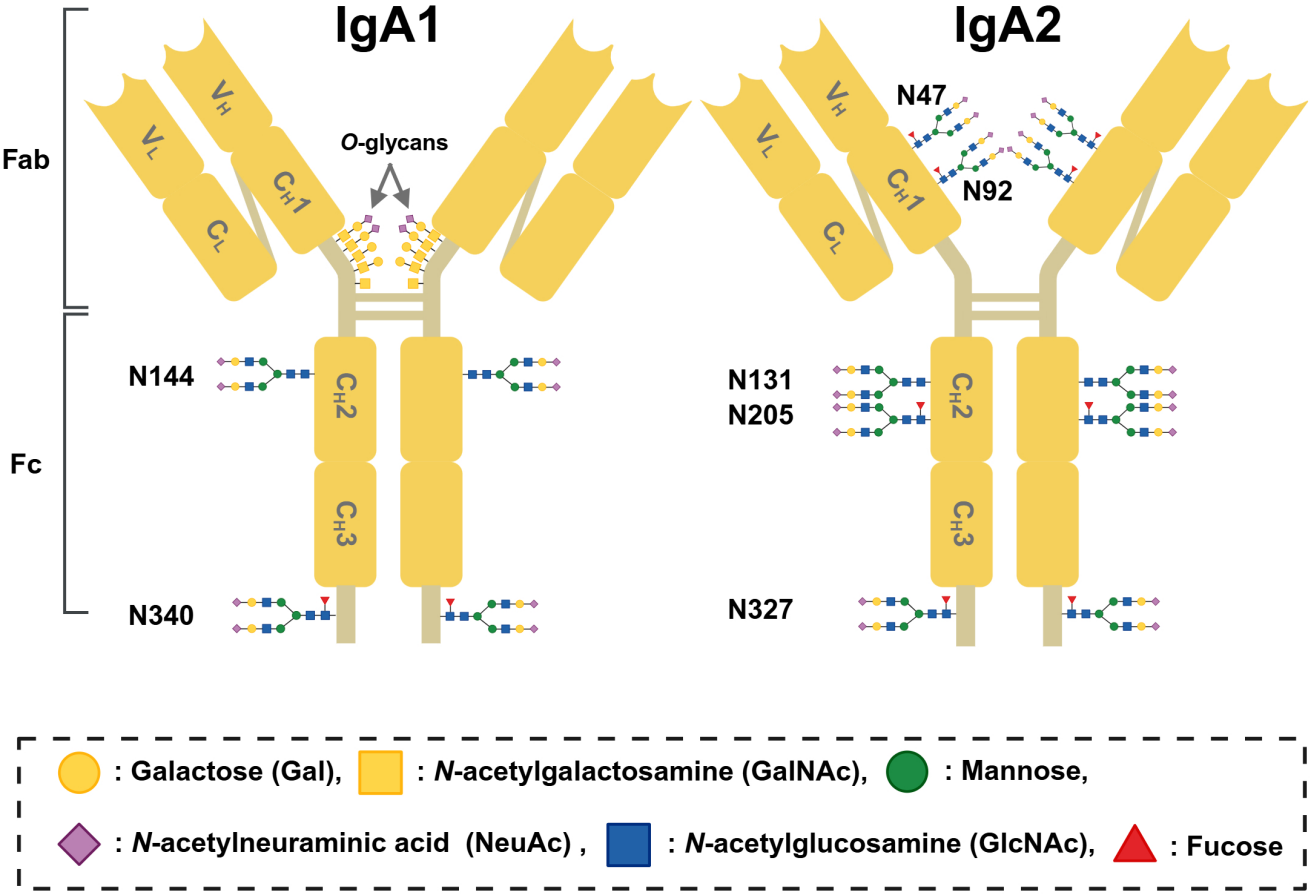
Schematic structure of human IgA1 and IgA2. Human IgA consists of two heavy (α) chains and two light chains (λ or κ). Each heavy chain contains one variable domain (V_H_) and three constant domains (C_H_1–C_H_3). The fragment antigen-binding (Fab) region comprises one light chain (variable light domain, V_L_; constant light domain, C_L_) and the *N*-terminal portion of one heavy chain (V_H_ and C_H_1). The fragment crystallizable (Fc) region is formed by C_H_2 and C_H_3. IgA1 possesses a long, proline-rich hinge region containing three to six *O*-glycans. Both IgA1 and IgA2 carry two *N*-glycosylation sites in the Fc region (N144/N131 and N340/N327, respectively). IgA2 contains two additional *N*-glycosylation sites in each chain (N47, N92, and N205), among which N92 is incorporated into an *N*-glycosylation consensus sequence only in IgA2m (2) and IgA2n allotypes.

IgA consists of two heavy (α) chains and two light chains (λ or κ). In humans, IgA has two subclasses, IgA1 and IgA2, due to the constant parts of two distinct α chains, encoded by the *Cα1* and *Cα2* genes (56). IgA1 is the predominant form in the serum and produced by both the systemic and mucosal compartments. In contrast, IgA2 is largely restricted to the gastrointestinal tract and tends to be more abundant than IgA1 in the distal intestine, saliva, and colostrum (115). Serum IgA2 represents 11–23% of total IgA (115). The most notable structural distinction between IgA1 and IgA2 is their glycosylation pattern. IgA1, but not IgA2, has 3–6 *O*-glycans in its proline-rich hinge region of the heavy chains. Each α1 chain of IgA1 also contains two *N*-glycans at asparagine (Asn)^144^ and Asn^340^. IgA2 lacks *O*-glycans but has four *N*-glycans on each α2 chain (or five in the IgA2m (2) allotype) at Asn^47^, Asn^131^, Asn^205^, and Asn^327^ (116–118) (**Figure 5**). IgA molecular forms include monomeric, dimeric, and higher polymeric forms, and IgA in secretions has attached secretory component (SIgA). In humans, circulating IgA primarily consists of monomeric IgA (mIgA), with only 10–20% present as dimeric IgA (dIgA) or pIgA (119). In contrast, mice reportedly exhibit comparable levels of monomeric and dimeric IgA under physiological conditions (120).

### 3.2 Features of circulating IgA in IgAN

Elevated blood IgA levels in patients with IgAN compared to those in healthy individuals have been confirmed in both Western and Asian populations (121–123). Furthermore, circulating IgA1 levels are elevated in patients with IgAN (123–127), which is supported by experimental results showing an increase in the proportion of IgA1 plasma cells in the bone marrow (123, 128). In addition, this increase is restricted to the IgA1 subclass with a predominance of λ light chains (126, 127). Previous renal biopsy data have shown that predominance of λ light chain deposition over κ is a unique characteristic to IgAN compared with other glomerular diseases (129–131). Moreover, λ predominance on immunohistochemistry is associated with increased endocapillary hypercellularity; however, its relationship with clinical prognosis remains unclear (132).

The proportion of pIgA in the circulation of patients with IgAN remains unresolved. Three studies have shown a preferential increase in pIgA (133–135). However, other investigators found no increase (128, 136, 137) or an increase with a concomitant increase in mIgA of similar magnitude (138). These results suggest that selectively elevated levels of pIgA are not a uniform feature in IgAN. Conversely, some studies indicated that pIgA has binding affinity for mesangial cells and promotes mesangial proliferation (139, 140), whereas other studies suggested that such stimulatory IgA is bound in immune complexes (140–144). Therefore, recurrent transient elevations in pIgA1-containing immune complexes in the circulation may contribute to IgAN progression.

### 3.3 Glycosylation features of circulating IgA in patients with IgAN

As previously noted, IgA1, a subclass predominantly elevated in the circulation of patients with IgAN, is a heavily glycosylated Ig containing both *N*- and *O*-glycans. The pathogenic significance of aberrant IgA1 *O*-glycosylation in IgAN was initially identified through differential lectin reactivity patterns observed in serum IgA1 from patients with IgAN compared to that in healthy individuals (145). IgA isolated from patients with IgAN exhibited increased reactivity with *Helix aspersa* agglutinin (HAA; a lectin that specifically recognizes the terminal *N*-acetylgalactosamine (GalNAc) of *O*-glycans), whereas not with lectins recognizing *N*-acetylglucosamine (GlcNAc) or galactose (Gal) on *N*-glycans, such as *Triticum vulgaris* agglutinin (TV; binds terminal GlcNAc) and *Erythrina crystagalli* agglutinin (EC; binds terminal D-Gal) (145). These findings indicate that patients with IgAN exhibit increased hypogalactosylated *O*-glycan levels in the hinge region of IgA1. The higher binding affinity of IgA from patients with IgAN to HAA compared to that of IgA from healthy individuals and disease controls has been validated across large cohorts of both Caucasian and Asian populations (122, 146). Furthermore, the binding affinity of HAA to pIgA is approximately four-fold higher than that to mIgA, suggesting that pIgA exhibits a lower degree of galactosylation (139). A rat monoclonal antibody KM55 was developed using immunization of a KLH-linked synthetic IgA1 hinge-region glycopeptide with 5 GalNAc residues (147). IgA1 recognized by KM55 is elevated in the circulation and in glomerular deposits of patients with primary and secondary IgAN (148, 149).

Recent studies employing high-resolution mass spectrometry (HRMS) have further characterized the molecular features of IgA1 *O*- and *N*- glycans in patients with IgAN. Dotz et al. reported that multiple glycosylation changes in IgA1 and IgA2, including alterations in galactosylation, sialylation, bisection, fucosylation, biantennary *N*-glycans, and *O*-glycan sialylation, are associated with IgAN, although galactose deficiency in IgA1 hinge region *O*-glycans was not observed as a characteristic feature in their study (150). Specifically, galactosylation and sialylation of *N*-glycans at Asn^144^ and sialylation of *O*-glycans in IgA1were lower in patients with IgAN than in healthy volunteers, whereas *N*-glycan bisection and sialylation at Asn^144^, biantennary *N*-glycan abundance at Asn^340^, and *N*-glycan fucosylation at Asn^340^ glycans were elevated in patients with IgAN. Yu et al. compared the *O*-glycosylation profile of pIgA1 among patients with crescentic IgAN, non-crescentic IgAN, and healthy volunteers and demonstrated that the number of GalNAc residues, i.e., the number of *O*-glycans per IgA1 HR, was reduced in patients with IgAN, particularly those with crescentic IgAN (151). This decrease in GalNAc content has also been independently reported by three other research facilities analyzing circulating IgA1 (152–154). Furthermore, only the combination of immunosuppressive therapy and palatine tonsillectomy increased the number of GalNAc residues in the IgA1 HR and decreased fucosylation level at the Asn^340^ *N*-glycan site; these alterations were not observed with immunosuppressive or supportive therapy alone (155).

In parallel with these biochemical insights, several studies highlighted the racial and genetic factors associated with IgA1 *N*- and *O*-glycosylation. Circulating Gd-IgA1 levels are higher in Caucasians than in Asians, among both healthy individuals and patients with IgAN (154, 156). The mean numbers of Gal and GalNAc residues attached to the IgA1 HR are lower in Caucasians than in Asians (154), which is consistent with miRNA upregulation regulating *O*-glycosylation-related enzymes in Caucasian populations (157). GWAS analyses further demonstrated that genetic variations in *C1GALT1* (gene encoding core 1 β1,3-galactosyltransferase), *C1GALT1C1* (gene encoding C1GalT1 chaperone), and *GALNT12* (gene encoding *N*-acetylgalactosaminyltransferase 12), the genes encoding enzymes involved in *O*-glycosylation, significantly influence circulating Gd-IgA1 levels (156, 158, 159). Additionally, a cohort study of genetically identical twins revealed that both *N*- and *O*-linked IgA1 glycosylation are strongly influenced by genetic factors, whereas *O*-glycosylation is more substantially affected by environmental factors than *N*-glycosylation (160).

### 3.4 Molecular size and glycosylation features of mucosal type of IgA

Polymeric IgA molecules are transported across the epithelium as dimers and higher-order polymers. Although the IgA oligomerization mechanism is poorly understood, the J-chain-stabilized dimer core serves as a structural unit for higher-order IgA polymers, in agreement with earlier findings that tetrameric IgA dissociates into a J chain-linked dimer and two monomers under mildly reducing conditions (161).

Glycosylation is a crucial post-translational modification that significantly influences protein tertiary structure and function. IgA glycosylation patterns vary depending on the secreted tissue. Site-specific glycosylation studies of immunoglobulins in non-plasma human samples have been largely restricted to the cerebrospinal and synovial fluids for IgG (162, 163) and colostrum for IgA (164, 165). Glycosylation of saliva-derived IgG and IgA has been examined using lectin-binding assays (166, 167); it was noted that inflammatory conditions can shift toward hypogalactosylated glycoforms of IgG (167). Comparative analysis of *N*- and *O*-glycosylation of IgA from saliva vs. plasma revealed substantial differences in the glycosylation profiles of IgA1, IgA2, and the J chain. Saliva-derived proteins exhibit increased levels of bisecting GlcNAc and reduced galactosylation and sialylation in *N*- glycans compared to the plasma-derived IgA (117). Regarding IgA1 *O*-glycans, salivary IgA1 showed a trend toward 1.4-fold lower sialic acid content relative to plasma IgA1, although this difference was not statistically significant. The abundance of GalNAc and Gal residues were also nearly identical between two groups (117).

Although the detailed molecular differences between mucosal IgA and circulating IgA have not been fully elucidated, these findings suggest that mucosal IgA tends to have a higher molecular weight and distinct *N*- and *O*-linked glycan structures than circulating IgA, implying a possible relationship with the molecular characteristics of IgA that is elevated in the circulation of patients with IgAN.

## 4 Factors influencing mucosal IgA immune system in patients with IgAN

As described above, IgA production is initiated in MALT cells, primarily within the gut, where B cells are primed by the microbiota and subsequently undergo CSR and SHM before differentiating into plasmablasts and plasma cells. Circulating IgA implicated in IgAN pathogenesis may differ from normal serum IgA, including glycosylation patterns. The induction of aberrant IgA arises from abnormalities in the mucosal microbiota and/or MALT and this possibility has been explored from multiple perspectives. In this section, we review mucosal immune abnormalities reported in patients with IgAN, focusing on the microbiota, antimicrobial peptides, TLR-mediated stimulation, alterations in cytokine profiles, and dysregulated B-cell homing. Notably, there are genetic effects, as evidenced by results from GWAS.

### 4.1 Gut and oropharyngeal dysbiosis

As previously noted, secretory IgA secreted contributes to the maintenance of homeostasis of the intestinal and oral microbiota by balancing immune exclusion vs. inclusion (14). “Gut dysbiosis” refers to the disruption of the microbiome balance characterized by altered microbial composition that can act as a strong trigger for immune modulation in the MALT and enhanced Gd-IgA1 production. Therefore, numerous studies have analyzed the differences in bacterial flora between patients with IgAN and healthy volunteers (**Supplementary Tables 1**, **2**).

The involvement of gut microbiota has been further substantiated by demonstrating that IgA reactive with components of the gut microbiota was increased in the serum of BAFF-transgenic mice. This elevated IgA, which is under-glycosylated and polymeric, drives glomerulonephritis, which is characterized by IgA deposition. Importantly, this high IgA syndrome does not develop in the absence of intestinal microbial colonization, i.e., in germ-free mice, demonstrating that the presence of gut bacteria is essential for IgA induction (168). This evidence supports the role of intestinal microbiota in the gut–kidney axis.

Angelis et al. showed that some traits of the gut microbiota varied significantly between patients with non-progressive IgAN, progressive IgAN, and healthy volunteers in an Italian cohort (169). They demonstrated that the fecal microbiota in patients with IgAN was altered, showing a tendency toward decreased levels of probiotic bacteria, such as *Lactobacillus* and *Bifidobacterium*, along with an increase in LPS-producing taxa, including *Sutterellaceae* and *Enterobacteriaceae*. Additionally, they showed that patients with progressive IgAN had high proportion of *Ruminococcaceae*, *Lachnospiraceae*, *Eubacteriaceae*, and *Streptococcaceae.*

Multiple studies comparing the gut microbiota of patients with IgAN and healthy volunteers identified several bacterial genera that exhibit consistent changes (**Supplementary Table 1**).

*Bifidobacterium* and *Lactobacillus* have recently attracted attention as probiotics and are gut microbial groups reduced in patients with IgAN (169–172). Members of the genus *Bifidobacterium* are among the first bacteria to colonize the human intestinal tract, a process promoted by the bifidogenic effects of specific oligosaccharides derived from human breast milk. Although the total abundance of *Bifidobacterium* decreases with age, it remains relatively stable throughout adulthood (2–14%) (173). *Bifidobacterium* species exert various health-promoting effects, notably in maintaining intestinal integrity as they support expression of tight junction proteins such as occludin, zonula occludens (ZO)-1, and claudinin by epithelial cells (174, 175). Furthermore, extracellular vesicles derived from *Bifidobacterium longum* play an anti-inflammatory role by improving the intestinal barrier, modulating immune cell differentiation, and promoting short-chain fatty acid production (176). *Lactobacillus* comprises a large heterogeneous group of gram-positive, facultative anaerobic bacteria and is one of the most widely used probiotics. These bacteria also contribute to the maintenance of epithelial barrier (177).

Relative enrichment of *Escherichia–Shigella* in patient with IgAN has been reported by several investigations (171, 172, 178–183). Zhao et al. investigated 127 treatment-naïve patients with IgAN and 127 matched healthy volunteers (181). The group was divided into discovery and validation cohorts. *Escherichia–Shigella* proportion increased in the gut microbiota before treatment, which improved in patients who achieved remission after six months of immunosuppression. In this study, *Escherichia–Shigella* abundance was positively correlated with serum creatinine, proteinuria, and Oxford pathologic classification severity and negatively correlated with serum albumin and eGFR (181). In addition, Zhu et al. demonstrated that serum levels of KM55-reactive IgA positively correlated with bacterial taxa enriched in IgAN, including *Shigella*, and associated with TLR4, BAFF, APRIL, LPS, and intestinal damage indicators (D-LAC and sICAM-1) (182). Furthermore, mice colonized with gut microbiota from patients with IgAN exhibited the activation of intestinal TLR4-mediated B-cell-stimulating pathways.

Gleeson et al. reported that *Akkermansia muciniphila* increased in the gut microbiota of patients with IgAN (184). Some bacteria can degrade and utilize mucin layer covering the gastrointestinal tract. Mucins contain *O*-glycans similar to those found in the hinge region of IgA1 (185) and *A. muciniphila*-expressed β-galactosidases can remove galactose residues from *O*-glycans and *N*-glycans. *A. muciniphila* can deglycosylate human IgA1 *in vitro* and induce IgA glomerular deposition in a humanized mouse model (α1^KI^-CD89^tg^ mice).

While many studies have focused on the gut microbiome in IgAN, emerging evidence suggests that oropharyngeal microbiota may also contribute to IgAN pathogenesis. Several studies have examined microbial communities in the oropharyngeal region, including those in the saliva and tonsils (**Supplementary Table 2**). Misaki et al. reported that the presence of *Streptococcus mutans* harboring the *cnm* gene in the saliva correlates with dental caries status and urinary protein levels in patients with IgAN (186). Yamaguchi et al. conducted an IgA-SEQ analysis on tonsillar tissues from patients with IgAN and compared the results with those in patients with recurrent tonsillitis cases (187). Their analysis revealed that *Porphyromonas* and *Prevotella* (phylum *Bacteroides*) were coated with IgA in the tonsillar crypts of patients with IgAN. Furthermore, Ig repertoire sequencing of the tonsillar IgA heavy chain demonstrated increased expression of *IGH* variable gene VH3–30, which correlated with the IgA-coating enrichment score of *Bacteroidetes*. In contrast, high-throughput 16S ribosomal RNA (rRNA) gene sequencing of palatine tonsils at the same institution failed to differentiate IgAN from recurrent tonsillitis, suggesting that IgA-bound microbiota, rather than the overall microbial composition, may be more relevant to disease pathogenesis (188).

Currie et al. performed a 16S rRNA analysis of tonsillar microbiota obtained from a cohort comprising multiple racial groups, including Pacific, East Asian, South Asian, Caucasian, African American, and others (189). They demonstrated an increased carriage of the genus *Neisseria* and elevated levels of *Neisseria*-targeted serum IgA in patients with IgAN. They also showed that the colonization *Neisseria* in humanized Carcinoembryonic antigen-related cell adhesion molecule (CEACAM)-1 transgenic BAFF-transgenic mice resulted in augmented systemic levels of *Neisseria*-specific IgA. Additionally, anti-*Neisseria* IgA-secreting cells were observed in the kidneys of mice. These findings suggest that cytokine-driven aberrant mucosal immune responses to oropharyngeal pathogens such as *Neisseria*, may contribute to IgAN immunopathogenesis.

### 4.2 Alteration in antimicrobial peptides

Bacteria produce antibacterial proteins, called bacteriocins, amphipathic molecules with narrow spectrum of activity that target particular bacterial pathogens (190). Conversely, antimicrobial peptides (AMPs) synthesized by complex life forms, including animals, insects, and plants, target a wide spectrum of pathogens and their biological significance extends far beyond microbial killing (191, 192).

In humans, the epithelial cells lining the skin, gastrointestinal tract, and respiratory tract generate several AMPs that play a crucial role in the defense against constant microbial exposure from the environment, while simultaneously preserving the remaining host microbiota (193). Defensins, cathelicidins, C-type lectins, galectins, ribonucleases, and S100 proteins are representative examples of AMP families in the gut and skin, and additional types have been detected in other specialized tissues, such as the oral cavity, nasal mucosae, eye, lung, and reproductive tract (193).

Genetic studies indicated that defensins, a major family of AMPs expressed in the small and large intestines, skin, and respiratory tract, are involved in IgAN pathogenesis. Human defensins consist of two major classes according to their cysteine disulfide connectivity: α-defensins and β-defensins. The α-defensin family has six members, which further separated into the human neutrophil peptides (HNPs, α-defensins 1–4), and human α-defensins (HD) HD5 and HD6, which are released by the intestinal Paneth cells in to the gut lumen (194, 195). The single nucleotide polymorphism (SNP) rs2738048 located in 8p23, which encodes α-defensin (*DEFA*), was identified as a susceptibility locus for IgAN through GWAS in three independent cohorts of Han Chinese, comprising 4,137 cases and 7,734 controls (196). This association was further validated in a Han Chinese cohort (197). New genome-wide significant signals in the *DEFA* locus at 8p23, rs10086568, and rs9644778 have been detected in individuals of ancestries (24). Moreover, another SNP rs2075836 in *DEFA1/4*, was detected in a GWAS of an international cohort consisting of 46.2% and 54.8% individuals of East Asian and European ancestries, respectively (198).

Copy number variations (CNVs) is a key form of genetic diversity in the human genome and can significantly influence physical traits by altering the number of gene copies, interfering with gene-coding regions, or affecting gene regulation over long distances (199). Ai et al. investigated three independent CNVs within the locus: *DEFA1A3*, *DEFA3*, and a non-coding deletion variant (*211bp*) in Chinese patients with IgAN and healthy volunteers (200). They demonstrated that a low total copy number of the three variants was associated with increased IgAN risk and significantly associated with renal dysfunction and elevated serum IgA1 and Gd-IgA1 levels (the latter measured by lectin assay) in patients with IgAN. Significant associations between *DEFA1A3* and *DEFA3* were also observed in the Caucasian cohort, mirroring the genetic effects found in the Chinese cohort, while *211bp* was rare in the Caucasian population, with consistent genetic effects as in the Chinese cohort (200). Serum and urine concentrations of HNP1–3 were elevated in patients with IgAN compared to those in healthy volunteers, independent of the copy-number distributions of *DEFA1A3* CNVs. On the other hands, neutrophils from healthy individuals produced more HNP1–3 than those from patients with IgAN when stimulated by LPS (200).

The mechanisms by which *DEFA* genotypes and *DEFA1A3* copy number variations contribute to the development or progression of IgA nephropathy remain unclear. Defensins not only mediate microbial killing but also exhibit chemotactic activity and modulate TLR responsiveness, indicating complex roles in immune regulation within MALT (193, 201, 202). Recent large-scale plasma proteomic studies have identified DEFA1 and DEFA1B as factors associated with Crohn’s disease (203). Elevated circulating HNP1–3 levels in IgAN patients suggest underlying MALT inflammation. Thus, further studies are needed to clarify how this inflammation contributes to increased circulating Gd-IgA1 and IgA levels, including potential links to the migration patterns and homing receptors of IgA-ASCs (204, 205).

### 4.3 TLR stimulation

TLRs are pattern-recognition receptors that play key roles in the innate immune system to defend against pathogens and cancer by sensing and responding to the structure-conserved molecules of PAMPs as well as the endogenous ligands released from damaged cells (damage-associated molecular patterns, or DAMPs) (206). Ten TLRs have been identified in humans (TLR1–TLR10) and 12 in mice (TLR1–TLR9 and TLR11–TLR13), with TLR1–TLR9s being conserved in both species (207). TLR10 is not functional in mice due to a mutation creating a stop codon, but human TLR10 collaborates with TLR2 to recognize ligands from *Listeria monocytogenes* (208) and sense influenza A viral infection (209). TLR10 binds HIV-1 gp41 protein, but its biological functions have not been fully elucidated (210) and, thus, it remains an orphan receptor.

**Figure 6:**
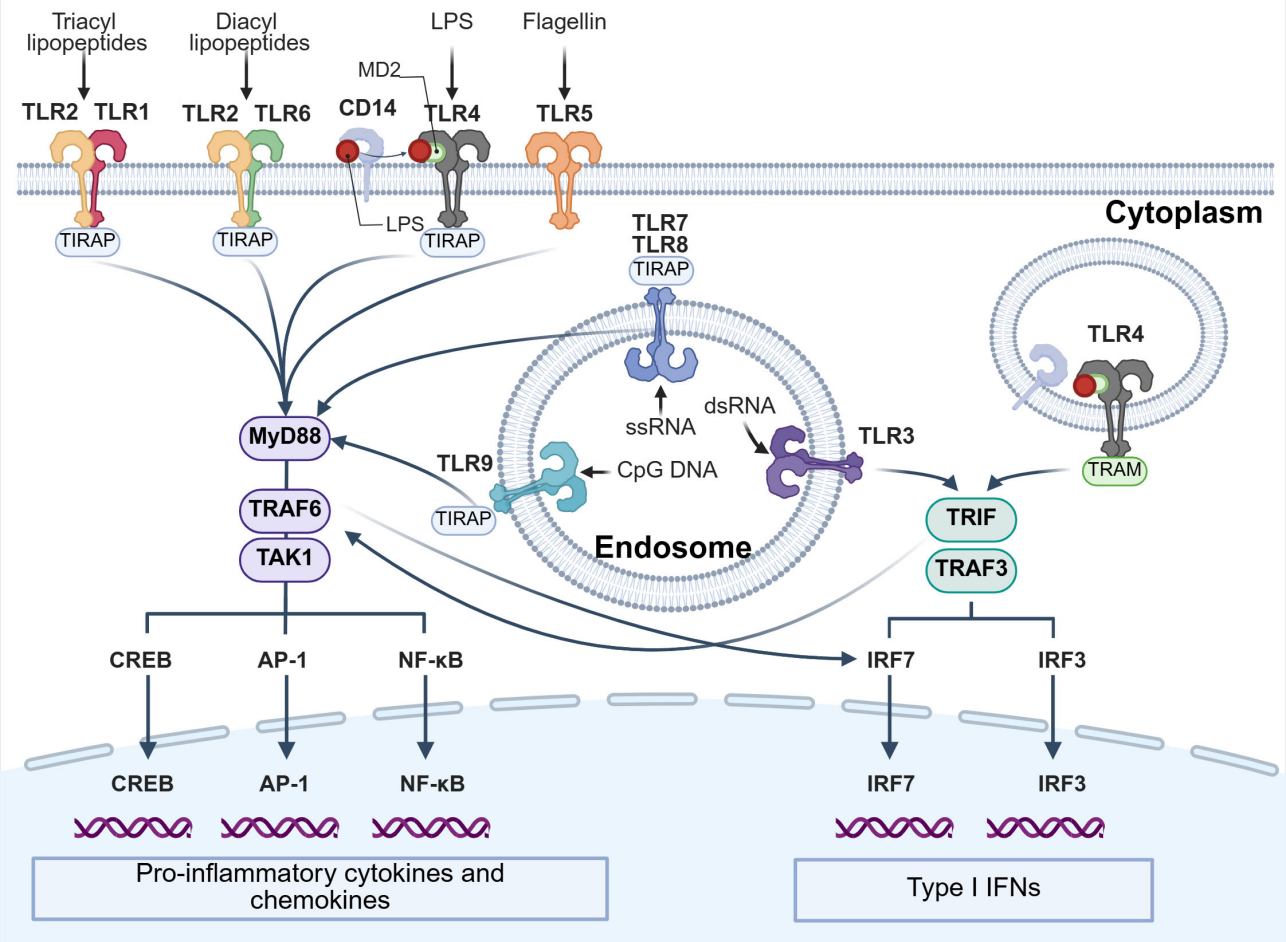
The combination of adapter proteins of each TLRs and its downstream pathway. This figure summarizes the human TLRs (TLR1–TLR9) whose ligands and biological functions have been well defined. TLRs are classified into two subfamilies based on their localization: cell-surface TLRs (TLR1, TLR2, TLR4, TLR5, and TLR6) and endosomal TLRs (TLR3, TLR7, TLR8, and TLR9). Individual TLRs interact with different combinations of adapter proteins and activate various transcription factors such as CREB, NF-κB, AP-1, and IRFs that induce the production of pro-inflammatory cytokines and chemokines, and IFNs via two different pathways: MyD88- and TRIF-dependent pathways. AP-1, activating protein-1; CpG, cytidine-phosphate guanosine; CREB, cyclic AMP-responsive element-binding protein; dsRNA, double-stranded RNA; IFN, interferon; IRF, interferon regulatory factor; LPS, lipopolysaccharide; MD2, TLR4 co-receptor myeloid differentiation factor2; MyD88, myeloid differentiation primary response protein 88; NF-κB, nuclear factor-κB; ssRNA, single-stranded RNA; TAK1, TGF-β-activated protein kinase 1; TIRAP, Toll/interleukin 1 (IL-1) receptor domain containing adaptor proteins; TRIF, TIRAP-inducing IFN-β; TLR, Toll-like receptor, TRAF, tumor necrosis factor receptor-associated factors; TRAM, TRIF-related adaptor molecule.

TLRs are classified into two subfamilies based on their localization: cell-surface (TLR1, TLR2, TLR4, TLR5, TLR6, and TLR10) and intracellular (TLR3, TLR7, TLR8, TLR9, TLR11, TLR12, and TLR13) TLRs (211). Individual TLRs engage different adaptor proteins and activate various transcription factors, including cyclic AMP-responsive element-binding protein (CREB), nuclear factor (NF)-κB, activating protein-1 (AP-1), and IFN regulatory factors (IRFs), through myeloid differentiation primary response protein 88 (MyD88)-dependent or Toll/interleukin 1 (IL-1) receptor domain containing adaptor proteins (TIRAP)-inducing IFN-β (TRIF)-dependent pathway (**Figure 6**) (206). All TLRs except TLR3 signal through the MyD88-dependent pathway, whereas TLR3 exclusively uses the TRIF-dependent pathway (212). In the MyD88-dependent pathway, TNF receptor-associated factors 6 (TRAF6)-mediated TGF-β-activated protein kinase 1 (TAK1) activation activates CREB, AP-1, and NF-κB. MyD88 also activates IRF5 and IRF7 downstream of TLR7, TLR8, and TLR9 signaling in pDCs (206). In the TRIF-dependent pathway, TRIF interacts with TRAF3 and TRAF6 and subsequently activates IRF3, IRF7, CREB, AP-1, NF-κB, and IRF5.

Each TLR is activated by a distinct ligand set (**Figure 6**). TLR4 is activated by LPS via TLR4 co-receptor myeloid differentiation factor2 (MD2) and transmits signals for the early-phase activation of NF-κB via Myd88-dependent pathway. The TLR4–MD2–LPS complex is also internalized and retained in the endosome, where it triggers signal transduction by recruiting the TRIF-related adaptor molecule (TRAM) (207). For these LPS signaling, CD14 plays a critical role. CD14 is expressed in macrophages and in other myelomonocyte lineages, including monocytes, neutrophils, and dendritic cells (213). The acylated moiety of lipid A in LPS is recognized by CD14 and LPS is transferred to the complex of TLR4 and MD2. LPS binding CD14 also plays a key role in endocytosis of TLR4 and following TRIF-dependent pathway (213). TLR2 forms a heterodimer with either TLR1 or TLR6, which recognizes triacyl and diacyl lipopeptides, respectively, and activates the MyD88 pathway via TIRAP. TLR5 recognizes flagellin, which is the main structural protein in the bacterial flagellum, and activates the MyD88 pathway (206, 207, 212). Intracellular TLRs primarily recognize nucleic acids derived from pathogens or self-nucleic acids under disease conditions. TLR3 recognizes double-stranded RNA and TLR7 and TLR8 recognize single-stranded RNA fragments, while TLR9 recognizes single-stranded DNA containing unmethylated CpG motifs from bacteria or viruses (206, 207, 212).

TLR9 and TLR7 may contribute to IgAN pathogenesis. Suzuki et al. demonstrated that TLR9/MyD88 pathway activation contributes to IgAN progression in ddY mice, that spontaneously develop the disease (214). ddY mice were stratified into groups with severe and mild glomerulonephritis (GN), and a GWAS was conducted to identify the susceptibility loci associated with renal injury progression. The *D9MIT216* marker locus, located in close proximity to MyD88-encoding gene, was identified as a susceptibility locus. The Splenic transcriptional levels *of TLR9* and *Myd88* were significantly higher in ddY mice maintained under conventional conditions than in those housed under specific pathogen-free (SPF) conditions, whereas *TLR2* and *TLR4* expression levels were comparable between the groups. Notably, *TLR9* and *MyD88* transcript levels of spleen increased with the severity of renal injury.

Elevated TLR9 expression has also been reported in the palatine tonsils of patients with IgAN (215) and some CD19^+^ B cells or pDCs express TLR9 (216). CpG-oligodeoxynucleotide (CpG-ODN), a ligand for TLR9, induces APRIL expression in CD19^+^ B-cells (215). Patients with high TLR9 expression in the palatine tonsils showed good therapeutic outcomes after tonsillectomy with steroid plus therapy (216). Furthermore, Fukao et al. recently demonstrated that the pDC proportion in palatine tonsils was significantly higher in patients with IgAN than in those with chronic tonsillitis and that its frequency correlated with APRIL, BAFF, and TLR9 expression in tonsillar mononuclear cells (MNC) (217). Production of IgA by cultured tonsillar MNCs increased in the presence of pDCs.

Genetic analysis revealed that specific TLR9 genotypes, CC or CT genotypes in rs352139 and TT genotypes in rs352140, were associated with an increased risk disease progression for Japanese patients with IgAN (214). In particular, patients with TT rs352140 genotypes show severe pathological damage and poor therapeutic outcomes after tonsillectomy with steroid plus therapy (216).

Involvement of TLR7 in IgAN pathogenesis has also been suggested. TLR7 recognizes synthetic imidazoquinoline-like molecules and guanosine analogs such as loxoribine, single-stranded RNA (ssRNA) derived from human immunodeficiency virus type 1 (HIV-1), vesicular stomatitis virus (VSV), influenza virus, and certain siRNAs (218). Recent reports about macroscopic hematuria in patients with IgAN after mRNA-based COVID-19 vaccination suggest the potential involvement of TLR7 as RNA-sensing pathways in exacerbation of IgAN. Zhang et al. recently reported that the relative *TLR7* and *GALNT2* mRNA levels in CD19^+^ B cells from peripheral blood were higher in patients with IgAN than in healthy volunteers. Thus, TLR7 may promote Gd-IgA1 production via the TLR7–GALNT2 axis in IgAN (219). Lee et al. demonstrated that the intranasal administration of imiquimod, a ligand of TLR7, and CpG-ODN, a ligand of TLR9, elevated the serum levels of aberrantly glycosylated IgA and induced glomerular IgA deposition and proteinuria in female ddY mice (220). These effects were abrogated by co-administration of hydroxychloroquine (HCQ), a known TLR signaling inhibitor. They also showed that TLR7 expression in the tonsils was elevated in patients with IgAN and positively correlated with the expression of APRIL, which is involved in production of aberrantly glycosylated IgA1. These findings suggest that nucleotide-sensing TLR9 and TLR7 play important roles in IgAN pathogenesis.

### 4.4 Alteration in cytokine profiles

*O*-glycosylation of IgA1 occurs in the Golgi apparatus of IgA-producing cells via a step-wise action of various glycosyltransferases, and *N*-glycosylation of IgA occurs sequentially in the endoplasmic reticulum and Golgi apparatus. These processes depend on specific enzymes and their expression that can be influenced by various cytokines.

In IgA1, *O*-glycosylation in the hinge region is initiated by the attachment of GalNAc to a serine or threonine residue catalyzed by UDP-*N*-acetylgalactosaminyltransferases (GalNAc-Ts), followed by the addition of Gal by C1GalT1. Production of folded active C1GalT1 depends on a specific molecular chaperone, Cosmc (C1GalT1C1). Furthermore, *N*-acetylneuraminic acid (NeuAc) is attached to Gal and GalNAc by α2,3-sialyltransferase (ST3Gal1) and α2,6-sialyltransferase (ST6GalNAc2), respectively.

Yamada et al. demonstrated that IL-4 stimulation decreases the expression of C1GalT1 and its chaperone, Cosmc, in the IgA1^+^ B lymphoma cell line DAKIKI (221). Xiao et al. showed that TGF-β1 stimulation decreased the expression of *C1GALT1* and *COSMC* in the same cell line (222). Suzuki et al. demonstrated that IL-6 and IL-4 reduced IgA1 *O*-glycan galactosylation via decreased *C1GALT1* expression and activity and increased *ST6GALNAC2* expression and activity in immortalized IgA1-secreting cells derived from the circulation of patients with IgAN vs. healthy controls. Notably, prior sialylation of GalNAc by ST6GalNAc2 prevented subsequent galactosylation by C1GalT1. Furthermore, Inoue et al. showed that *GALNT2* expression was significantly lower in B cells stimulated with CD40L and IL-21, the cytokines associated with the TD pathways (223).

Recent GWAS analyses of patients with IgAN have suggested that APRIL, a member of the TNF superfamily encoded on chromosome 17, is a susceptibility gene for IgAN (196, 198). APRIL plays key roles in late-stage B-cell differentiation, IgA class switching, and generation and maintenance of antibody-secreting cells. APRIL is produced by various myeloid cell populations, including macrophages, DCs, monocytes, and polymorphonuclear cell types (e.g., neutrophils, eosinophils), and mucosal epithelial cells (86, 96, 224, 225). Another cytokine, BAFF, shares 48% sequence homology with APRIL, although it is encoded on a different chromosome (chromosome 13). Elevated serum APRIL levels in patients with IgAN were confirmed in two independent cohorts from Toronto and Alabama, whereas serum BAFF levels were comparable between patients and healthy volunteers (168). However, other studies reported elevated serum BAFF levels in patients with IgAN (226, 227). The tissues and cells responsible for the overexpression of these cytokines have not yet been fully elucidated. However, APRIL expression is higher in the tonsils of patients with IgAN than in those with chronic tonsillitis, and the APRIL-producing cells are differently localized. APRIL-producing cells are present in the tonsillar epithelium and outside cells, identified as neutrophils, in both patients with IgAN and chronic tonsillitis, whereas APRIL-producing cells are significantly increased in the GC of patients with IgAN (215). APRIL-producing cells in GC are CD19^+^ B cells, and APRIL expression is correlated with TLR9 expression. *In vitro* experiments demonstrated that TLR9 stimulation enhanced ARPIL expression. These findings highlight the importance of TLR9-induced APRIL overexpression in tonsillar GC B cells in IgAN pathogenesis. Makita et al. reported that TLR9 stimulation in human IgA-secreting cell lines increases KM55-reactive IgA production through APRIL and IL-6 expression upregulation (228).

### 4.5 Altered B-cell homing

Leukocyte trafficking is a combinatorial process where distinct sets of adhesion molecules and chemokines confer specificity.

Patients with IgAN have elevated levels of circulating CCR9^+^ β7-integrin^+^ regulatory B cells (226). CCR9, a chemokine receptor interacting with CCL25, is released from small intestine, and β7 integrin is a family of integrin beta chain and binds to α4 integrin, forming the α4/β7 heterodimer that interact with MAdCAM-1, a glycoprotein expressed by endothelial cells at venules of Peyer’s patches, intestinal LP, and mesenteric lymph nodes. Furthermore, circulating Gd-IgA1^+^ cells from IgAN express predominantly λ light chains and CCR10 and CCR9, which play an important role in homing to salivary glands, upper respiratory and digestive tracts, mammary glands, and bone marrow (102, 229). However, responsiveness to the chemokine signaling of these cells has not yet been investigated.

The potential importance of lymphocyte glycans in homing was suggested in seminal studies by Gesner and Ginsberg (230, 231). Murine studies using a B-cell-specific knockout of Cosmc, a specific molecular chaperone for C1GalT1, revealed that impaired *O*-glycan elongation on B cells may reduce their responsiveness to chemokine signaling during homing (232). Furthermore, GalNAc-T14, one of the GalNAc-Ts, knockout mice have elevated serum IgA levels and increased glomerular IgA deposition with aging. In addition, *GALNT14*-deficient B cells exhibit impaired homing to lymphoid tissues and a tendency toward lymphocyte retention in the peripheral blood (233). Thus, alterations in expression or activity or some glycosyltransferases may not only modify the glycan structures of IgA, but also influence IgA-producing B-cell homing.

## 5 Current trends in new therapies targeting MALT

According to the KDIGO 2025 guidelines for IgAN treatment, treatment options can be broadly divided into two categories: those that target IgAN-specific nephron loss drivers and those that address the generic responses to IgAN-induced nephron loss (234). Systemic glucocorticoids have been widely used in the past few decades. However, the results of the STOP-IgAN and TESTING randomized clinical trials indicate that the efficacy and safety of systemic corticosteroid therapy remain controversial (235, 236). In the TESTING trial, systemic corticosteroid therapy demonstrated a beneficial effect in reducing the composite primary endpoint, defined as ≥40% decline in eGFR, progression to kidney failure requiring renal replacement therapy, or kidney disease-related death. Nevertheless, the high-dose steroid group showed a high incidence of serious adverse events including infections (236). Therefore, the development of new therapeutic agents with less side effects is urgently required.

Recent advances in understanding IgAN pathophysiology have led to the development of numerous therapeutic agents targeting abnormalities in GALT and IgA-producing B-cell survival and differentiation pathways. This section provides an overview of the emerging therapies that target molecules related to gut-associated immune tissues, focusing on clinical trials conducted in recent years. A list of these clinical trials is summarized in **Table 2**. Detailed information, including clinical trial outcomes, is presented in **Supplementary Table 3**.

**Table 2:** Clinical trials targeting the mucosal immune system.

Drug name	Mechanism	Trial name	Intervention	Primary outcome	Comparison	Reference
a. Targeted-release budesonide						
Targeted-release budesonide	Targeted delivery of budesonide, a synthetic corticosteroid, to ileal Peyer’s patches to modulate local immune dysregulation and reduce Gd-IgA1 and IgA-IC production.	Phase 2b DB-RCT NEFFIGAN NCT01738035	8 or 16 mg QD of TRF-budesonide (Nefecon) for 9 months (n=51, 51 respectively)	Change from baseline in UPCR at 9 months	Placebo (n=51)	Fellstrom et al., 2017 (Lancet) (238) Wimbury et al., 2024 (Kidney Int) (240)
		Phase 3 DB-RCT NefigArd NCT03643965	16 mg QD of TRF-budesonide (Nefecon) for 9 months (n=182)	Part A: Ratio of UPCR at 9 months compared to baseline Part B: Time-weighted average of eGFR at 2 years	Placebo (n=182)	Barratt et al., 2023 (Kidney Int) (239) Lafayette et al., 2023 (Lancet) (22)
b. APRIL inhibitors						
Sibeprenlimab (VIS649)	A humanized IgG2 monoclonal antibody that binds to and neutralizes APRIL activity	Phase 2 DB-RCT ENVISION NCT04287985	2, 4, and 8 mg/kg IV QM of sibeprenlimab for 12 months (n=38, 41, and38)	Change from baseline in the 24-hour UPCR at 12 months	Placebo (n=38)	Mathur et al., 2024 (N Engl J Med) (241)
		Phase 3 DB-RCT VISIONARY NCT05248646 (Ongoing)	400 mg SC Q4W of sibeprenlimab for 26 doses (n=152)	Change from baseline in the 24-hour UPCR at 9 months	Placebo (n=168)	Perkovic et al., 2025 (N Engl J Med) (242)
Zigakibart (BION-1301)	A humanized IgG4 monoclonal antibody that binds to and neutralize APRIL activity	Phase1/2 Open-label extension NCT03945318	Cohort 1) 450 mg IV Q2W transitioned to 600mg SC Q2W for total 124 weeks (n=10) Cohort 2) 600 mg SC Q2W for 124 weeks (n=30)	Safety data: Incidence of TEAEs	―	Kooienga et al., 2025 (Kidny Int) (243)
		Phase 3 DB-RCT BEYOND NCT05852938	600 mg SC Q2W for 104 weeks.	Change in UPCR from baseline at 40 weeks	Placebo	Under investigation
c. APRIL and BAFF inhibitors						
Atacicept (VT-001)	Human TACI–Fc fusion protein that binds and neutralizes BAFF and APRIL	Phase 2 DB-RCT JANUS NCT02808429	25, 75 mg SC QW (n=6, 5) for up to 72 weeks	Safety data: Incidence of TEAEs, AEs	Placebo (n=5)	Barratt et al., 2022 (Kidney Int Rep)
		Phase 2b DB-RCT ORIGIN 3 NCT04716231	After DB-RCT period for 36 weeks (Placebo, n=34; 25/75/150 mg SC QW, n=82), 113 patients entered the open-label extension period: (150 mg SC QW for 60 weeks)	Change from baseline in the 24-hour UPCR at 36 weeks	Placebo (n=34)	Lafayette et al., 2024 (Kidney Int) (244) Barratt et al., 2025 (JASN) (245)
		Phase 3 DB-RCT ORIGIN 3 NCT04716231	150 mg SC QW for 60 weeks (n=106)	Change from baseline in the 24-hour UPCR at 36 weeks	Placebo (n=97)	Lafayette et al., 2025 (N Engl J Med) (246)
Telitacicept (RC18)	Human TACI–Fc fusion protein that binds and neutralizes BAFF and APRIL	Phase 2 DB-RCT NCT04291781	160, 240 mg SC QW (n=16, 14) for 24 weeks	Change from baseline in 24-hour proteinuria at 24 weeks	Placebo (n=14)	Lv et al., 2023 (247) (Kidney Int Rep) Zan et al., 2024 (Kidney Int Rep) (248)
		Phase 3 TELIGAN NCT05799287	240 mg SC QW in phase A for a total of 39 doses and Q2W in phase B for a total of 32 doses	Change from baseline in 24-hour proteinuria at 39 weeks Annualized eGFR slope over 104 weeks	Placebo	Under investigation
Povetacicept (ALPN-303)	An engineered TACI–Fc fusion protein with enhanced potency	Phase 1/2 open-label RUBY-3 NCT05732402	80, 240 mg SC Q4W (n=21, 33) for 24 weeks (have the option to participate in additional 28-week and 52-week extension)	Safety: Incidence of TEAEs	―	Madan et al., 2026 (Kidney Int Rep) (250)
d. B-cell depleting agents						
Felzartamab	A fully human anti-CD38 monoclonal antibody	Phase 2a DB-RCT IGNAZ NCT05065970	1) In part 1: IV on 1,8,15,22,29,57,85,113, and 141 days 2-dose, 5-dose, 9-dose by body weight (<50 kg, 650 mg; >50–70 kg, 975 mg; >70–90 kg, 1300 mg; and >90 kg, 1625 mg) (n=12, 11, and 13) 2) In part 2: 9-dose (six Japanese patients)	Change from baseline in 24-hour UPCR at 9 months	Placebo (n=12)	Floege et al., 2025 (Kidney Int) (254)
Mezagitamab (TAK-079)	A fully human anti-CD38 monoclonal antibody	Phase 1B open-label NCT05174221	SC QW for 8 weeks, then Q2W for 16 weeks	Safety of drug based on TEAEs at 48, 96 weeks and 0–48 weeks	―	Under investigation
Bortezomib (Velcade)	Proteasome inhibition leads to toxic protein buildup and triggers apoptosis in myeloma cells.	NCT01103778	At 1.3 mg/m^2^, on days 1, 4, 8 and 11 (=1 cycle)	24-hour urine protein at 1year Number of patients with complete remission Partial response or no response at 1 year	―	Under investigation
e. Immunomodulatory drug						
Hydroxychloroquine sulfate	Raise the intra-lysosomal pH and consequently reduce MHC class II-mediated autoantigen presentation. Accumulate in endosomes and inhibits TLR signaling.	DB-RCT NCT02942381	Daily oral HCQ for 6 months (n=30): 0.2 g (2 tablets) twice daily for patients with eGFR > 60 mL/min/1.73 m^2^ 0.1 g (1 tablet) 3 times daily for patients with 45 < eGFR < 59 mL/min/1.73 m^2^ 0.1 g (1 tablet) twice daily for patients with 30 < eGFR < 44 mL/min/1.73 m^2^	Percentage change in proteinuria between baseline and 6 months	Placebo (n=30)	Liu et al., 2019 (Am J Kidney Dis) (258)

eGFR, estimated glomerular filtration rate; Gd-IgA1, galactose-deficient IgA1; IgA-IC, IgA-containing immune complexes; APRIL, A proliferation-inducing ligand; BAFF, B-cell activating factor; DB-RCT, double-blind randomized controlled trial; QD, once daily; Q2W, every 2 weeks; Q4W, every 4 weeks; IV, intravenous; SC, subcutaneous; UPCR, urine protein-to-creatinine ratio; TEAE, treatment-emergent adverse event; AE, adverse event; HCQ, Hydroxychloroquine; N.S, not significan.

### 5.1 A targeted-release budesonide formulation

Targeted-release of budesonide (Nefecon) is an oral capsule engineered to deliver the drug specifically to the distal ileum, a region rich in Peyer’s patches, within the GALT. It undergoes substantial first-pass metabolism in the liver, which helps to limit systemic exposure to glucocorticoids (237). Phase 2 and phase 3 trials demonstrated that Nefecon effectively reduced proteinuria in IgAN. The phase 2 placebo-controlled study (NEFFIGAN) in patients with urinary protein-to-creatinine ratio (UPCR) > 0.5 g/g showed a 24.4% reduction in proteinuria at 9 months with Nefecon (21.5% with 8 mg and 27.3% with 16 mg) (238). In the phase 3 trial (NefigArd), adults with UPCR ≥0.8 g/g or proteinuria ≥1 g/24h receiving Nefecon 16 mg/day for 9 months achieved 40.9% reduction in time-averaged UPCR between 12 and 24 months compared with the placebo group (*P* < 0.0001) and showed a smaller decline in time-weighted average eGFR over 2 years compared with placebo (−2.47 vs. −7.52 ml/min/1.73 m²; *P* < 0.0001) (22, 239). Analysis of the biochemical pathways through which Nefecon exerted its effects in patients enrolled in the NEFIGAN study revealed that Nefecon significantly reduced circulating KM55-reactive IgA, IgA/IgG-immune complexes, and SIgA levels (240). It also lowered circulating fatty acid binding protein 2 (FABP2), a marker released from the small-intestinal epithelial cells in response to mucosal injury. Interestingly, Nefecon further altered the levels of several small intestine-derived chemokines, including CCL11, CCL13, CCL19, CCL20, CXCL5, CXCL6, and CXCL13. In addition, the levels of BAFF, APRIL, and their receptor BCMA were reduced after treatment. These findings support the involvement of mucosal barrier function and B-cell responses in the distal ileal GALT in IgAN pathogenesis (240).

### 5.2 APRIL and/or BAFF inhibitors

Recently, multiple BAFF/APRIL inhibitors have been developed, and clinical trials for IgAN are ongoing. Among these, sibeprenlimab (VIS649), a humanized IgG2 monoclonal antibody that binds to and neutralizes APRIL, has received accelerated FDA approval and its use in clinical practice has begun. In the phase 2 study, adult patients with IgAN and at least 0.75 g/g Cr 24-hour UPCR (or ≧1.0 g/day urinary protein level) were randomized to receive three different intravenous sibeprenlimab doses (2, 4, and 8 mg/kg), comparing with placebo (241). The study showed 47.2%, 58.8%, and 62.0% reduction in 24-hour UPCR reduction from baseline in the 2, 4, and 8 mg/kg dose groups, respectively, and 20% reduction in the placebo group at 12 months. The eGFR decline over 12 months in all sibeprenlimab groups was lower than that in the placebo group, with changes of −2.7, 0.2, and −1.5 ml/min/1.73 m² for the 2-mg, 4-mg, and 8-mg doses, respectively, compared with −7.4 ml/min/1.73 m² with placebo. In the 4- and 8-mg sibeprenlimab groups, the serum IgA and Gd-IgA1 levels (the assay method is not reported) were reduced by approximately 65%, IgG by approximately 35%, and IgM by approximately 75%. Despite reductions in the serum IgA, IgG, and IgM levels in the sibeprenlimab groups, the incidence of infection did not increase with sibeprenlimab; moreover, Covid-19 occurred more frequently in the placebo group than in the sibeprenlimab groups. Furthermore, in a phase 3, multicenter, double-blind, randomized, placebo-controlled trial (VISIONARY), adults with biopsy-proven IgAN were assigned in a 1:1 ratio to receive subcutaneous sibeprenlimab 400 mg or placebo every 4 weeks for 100 weeks. Interim analysis at 9 months showed a significant reduction in 24-hour UPCR with sibeprenlimab (–50.2%) than the increase with placebo (2.1%) (242).

Zigakibart (BION-1301), another humanized IgG4 monoclonal antibody that binds to and blocks APRIL binding to BCMA and TACI, also demonstrated therapeutic efficacy against IgAN in a phase 1/2 open-label study. Subcutaneous administration of 600 mg zigakibart every two weeks in patients with IgAN showed 60% reduction in proteinuria and sustained eGFR stabilization at week 100. A notable decrease in hematuria was observed, as well as rapid and durable reductions in IgA, KM55-reactive IgA, and IgM levels, with a modest reduction in IgG (243). The results of the phase 3 BEYOND trial are highly anticipated.

Atacicept is a native human TACI–Fc fusion protein that binds to and neutralizes BAFF and APRIL, thereby modulating B-cell activity and reducing the circulating levels of KM55-reactive IgA, anti-Gd-IgA1 autoantibodies, and immune complexes. In a phase 2b study, 52% UPCR reduction from baseline was observed at 96 weeks in the atacicept group (subcutaneous administration of atacicept 150 mg every week for week 60) (244, 245). In the pre-specified interim analysis of phase 3 trial, once-weekly 150 mg atacicept administration to patients with IgAN reduced 45.7% UPCR from the baseline, compared with 6.8% reduction in the placebo group at week 36. Gd-IgA1 levels (the assay method is not reported) were reduced as early as week 4, and a decrease in dip-stick hematuria was observed at week 36 (246).

Telitacicept, another TACI–Fc fusion protein, neutralizes BAFF and APRIL and disrupts its interaction with TACI. Subcutaneous weekly 240 mg telitacicept administration substantially reduced proteinuria by 49% from baseline and notably impacted IgA, IgG, and IgM (247). In a subset of patients from a phase 2 trial, the levels of KM55-reactive IgA, IgG-IgA immune complex, and polymeric-IgA immune complex decreased in the telitacicept group, but circulatory C3a, C5a, or sC5b-9 levels did not change significantly during telitacicept treatment (248). Phase 3 trial (TELIGAN) is ongoing in China.

Povetacicept (ALPN-303), an engineered TACI-Fc fusion protein demonstrated strong inhibitory activity and favorable pharmacokinetic/pharmacodynamic profiles in preclinical models. In an in-human study, single doses of up to 960 mg were well tolerated by healthy adults and produced dose-dependent pharmacokinetics. BAFF and APRIL suppression persisted for several weeks, and doses ≥80 mg induced maximal pharmacodynamic effects, including reductions in ASCs and all Ig isotypes, notably KM55-reactive IgA. These findings support the clinical development of povetacicept for treating B-cell-mediated autoimmune diseases (249). The phase1/2 trial for IgAN and membranous nephropathy yielded favorable results, and phase 3 trial results are highly anticipated (250).

Findings from clinical testing of BAFF/APRIL inhibitors demonstrate that these cytokines are critically involved in IgAN pathogenesis.

### 5.3 CD38 inhibitors and proteasome inhibitors

Based on the current understanding of IgAN pathogenesis, several B-cell-depleting agents have been tested as treatments. B-cell-depleting therapy has demonstrated therapeutic efficacy against nephropathy caused by autoimmune diseases that produce autoantibodies, such as membranous nephropathy, lupus nephritis, and ANCA-associated vasculitis. Therefore, this therapy should also reduce Gd-IgA1 and anti-Gd-IgA1 antibodies, as well as provide renoprotective effects in IgAN.

Rituximab, a chimeric anti-CD20 monoclonal antibody, was evaluated in an open-label, multicenter study involving 34 patients with biopsy-proven IgAN (proteinuria >1 g/day, eGFR 30–90 ml/min/1.73 m^2^) (251). Although peripheral CD19^+^ B cells were effectively depleted, rituximab did not reduce proteinuria or improve eGFR over a one-year period compared to the placebo. Serum levels of total IgA, Gd-IgA1 (by lectin assay), and anti-Gd-IgA1 antibodies remained unchanged. As plasmablasts and plasma cells generally do not express CD20, these findings highlight the importance of targeting the cells responsible for secreting nephritogenic IgA and anti-Gd-IgA1 antibodies as a part of therapeutic strategies. This study also underscores the need for drugs capable of adequately penetrating tissues where these cells reside or are generated (252).

Based on these results, the therapeutic potential of CD38-targeting monoclonal antibodies for IgAN is currently being investigated. CD38 is strongly expressed in antibody-secreting plasmablasts and plasma cells, including long-lived plasma cells (253).

Mezagitamab (TAK-079) and felzartamab are fully humanized anti-CD38 IgG1 monoclonal antibodies that bind with high affinity to CD38 that have been tested in clinical trials. They showed encouraging early-phase data (stages 1b and 2a) (254). Felzartamab treatment for 3 months reduced proteinuria and a sustained reduction in proteinuria was observed for 18 months after completing treatment. Both treatments are moving forward to phase 3 testing.

Proteasome inhibitors, which are used to treat multiple myeloma by blocking proteasome activity and by preventing degradation of misfolded or damaged proteins, accumulate toxic proteins in myeloma cells, resulting in cellular stress and apoptosis. A pilot trial evaluated the effects of bortezomib, a proteasome inhibitor, in patients with IgAN. Three of the eight patients achieved complete remission, suggesting that bortezomib may be effective in selected cases of IgAN (255). Randomized controlled trials are required to clarify the efficacy of proteasome inhibitors.

### 5.4 Hydroxychloroquine

Hydroxychloroquine (HCQ), originally developed as an antimalarial agent, is widely used as an immunomodulatory drug for autoimmune diseases, such as rheumatoid arthritis and systemic lupus erythematosus. It shares the 4-aminoquinoline core structure with chloroquine but differs in the hydroxylation of its basic side chain and is characterized by extensive tissue distribution and a long half-life (256). Recent clinical studies have demonstrated that HCQ reduces proteinuria in patients with IgAN (257, 258).

HCQ enters and accumulates in lysosomes along the pH gradient, where it increases the intralysosomal pH and inhibits lysosomal enzymes. This function disrupts the degradation of extracellular cargo internalized by endocytosis or phagocytosis, as well as intracellular material processed through autophagy and can consequently reduce MHC class II-mediated autoantigen presentation (256).

HCQ can also accumulate in the endosomes and bind to the minor groove of double-stranded DNA. It inhibits TLR signaling by increasing the endosomal pH required for TLR processing and/or blocking ligand binding to TLR7 and TLR9. HCQ further suppresses the nucleic acid sensor cyclic GMP–AMP synthase (cGAS) by interfering with its interaction with cytosolic DNA. TLR and cGAS–stimulator of *IFN* gene (STING) pathway inhibition reduces the production of pro-inflammatory cytokines, including type I IFN (256).

The mechanisms by which HCQ exerts its therapeutic effects in IgAN are not fully understood. However, TLR7/9 stimulation in IgAN-prone ddY mice increased circulating levels of IgA and increased IgA glomerular deposition and proteinuria, whereas co-administration with HCQ prevented these kidney effects (220). TLR7/9 stimulation similarly elevated IgA, KM55-reactive IgA, APRIL, and IL-6 production by cultured human tonsillar mononuclear cells (220). These findings support the possibility that HCQ ameliorates IgAN by inhibiting TLR7/9 signaling, thereby reducing Gd-IgA1, APRIL, and IL-6 production (220). In addition to its TLR-mediated effects, HCQ may suppress MHC class II-dependent antigen presentation in antigen-presenting cells (256), thereby attenuating T-cell activation and reducing inflammatory cytokine production. These potential mechanisms underscore the need for further investigations into the role of HCQ in modulating IgAN pathophysiology.

## 6 Conclusion

In this review, we have highlighted fundamental findings demonstrating that IgA induction within MALT requires exposure to the commensal microbiota and that secretory IgA released into the intestinal lumen contributes to maintaining microbial homeostasis and, in turn, supports mucosal epithelium integrity and the underlying lymphoid structures. Studies analyzing the microbiota of patients with IgAN have consistently shown compositional differences compared with healthy individuals. These observations suggest that mucosal immune network dysbiosis and disruption may alter IgA-producing B-cell differentiation and maturation, thereby promoting nephritogenic IgA generation. Moreover, IgA secreted by these aberrant B cells may further perturb the microbiota, potentially creating a vicious cycle among IgA, microbiota, and MALT. However, the primary trigger responsible for disturbing MALT homeostasis and microbiota remains unidentified.

As summarized in this review, several therapeutic agents targeting MALT and B cells derived from them have been developed. Current therapeutic strategies targeting B cells, such as APRIL inhibitors, dual APRIL/BAFF inhibitors, CD38 monoclonal antibodies, and proteasome inhibitors, have attracted considerable attention and may suppress nephritogenic IgA-producing cell differentiation and survival. However, the long-term durability of therapeutic effects of these novel agents and adverse effects associated with prolonged administration remain unclear and need to be elucidated in ongoing and future clinical trials. Abnormalities in the maturation or long-term survival of IgA-producing B cells represent a vicious cycle, and eliminating the IgA-producing cells responsible for nephritogenic IgA could contribute to sustained therapeutic efficacy. However, if the fundamental problem lies in the aberrant niches that generate nephritogenic IgA1-producing cells, recurrence is anticipated.

Considering these possibilities, future research should focus on therapeutic approaches that directly modulate mucosal immune environments, such as normalizing cytokine and chemokine profiles, strengthening epithelial barrier integrity, enhancing antimicrobial peptide secretion, and correcting homing defects. Such efforts may broaden the therapeutic landscape and ultimately lead to comprehensive strategies for IgAN management.
